# Engineering surface framework TiO_6_ single sites for unprecedented deep oxidative desulfurization

**DOI:** 10.1093/nsr/nwae085

**Published:** 2024-03-06

**Authors:** Shen Yu, Zhan Liu, Jia-Min Lyu, Chun-Mu Guo, Xiao-Yu Yang, Peng Jiang, Yi-Long Wang, Zhi-Yi Hu, Ming-Hui Sun, Yu Li, Li-Hua Chen, Bao-Lian Su

**Affiliations:** Laboratory of Living Materials at the State Key Laboratory of Advanced Technology for Materials Synthesis and Processing, Wuhan University of Technology, Wuhan 430070, China; Laboratory of Living Materials at the State Key Laboratory of Advanced Technology for Materials Synthesis and Processing, Wuhan University of Technology, Wuhan 430070, China; Nanostructure Research Center, Wuhan University of Technology, Wuhan 430070, China; Laboratory of Living Materials at the State Key Laboratory of Advanced Technology for Materials Synthesis and Processing, Wuhan University of Technology, Wuhan 430070, China; Laboratory of Living Materials at the State Key Laboratory of Advanced Technology for Materials Synthesis and Processing, Wuhan University of Technology, Wuhan 430070, China; Laboratory of Living Materials at the State Key Laboratory of Advanced Technology for Materials Synthesis and Processing, Wuhan University of Technology, Wuhan 430070, China; Laboratory of Living Materials at the State Key Laboratory of Advanced Technology for Materials Synthesis and Processing, Wuhan University of Technology, Wuhan 430070, China; School of Chemistry, Chemical Engineering and Life Science, Wuhan University of Technology, Wuhan 430070, China; Laboratory of Living Materials at the State Key Laboratory of Advanced Technology for Materials Synthesis and Processing, Wuhan University of Technology, Wuhan 430070, China; Nanostructure Research Center, Wuhan University of Technology, Wuhan 430070, China; Laboratory of Living Materials at the State Key Laboratory of Advanced Technology for Materials Synthesis and Processing, Wuhan University of Technology, Wuhan 430070, China; Laboratory of Living Materials at the State Key Laboratory of Advanced Technology for Materials Synthesis and Processing, Wuhan University of Technology, Wuhan 430070, China; Laboratory of Living Materials at the State Key Laboratory of Advanced Technology for Materials Synthesis and Processing, Wuhan University of Technology, Wuhan 430070, China; Laboratory of Living Materials at the State Key Laboratory of Advanced Technology for Materials Synthesis and Processing, Wuhan University of Technology, Wuhan 430070, China; Laboratory of Inorganic Materials Chemistry, University of Namur, Namur B-5000, Belgium

**Keywords:** electrostatic interactions, framework hexa-coordinated Ti site, oxidative desulfurization, mesoporous materials, self-assembly

## Abstract

Catalytic oxidative desulfurization (ODS) using titanium silicate catalysts has emerged as an efficient technique for the complete removal of organosulfur compounds from automotive fuels. However, the precise control of highly accessible and stable-framework Ti active sites remains highly challenging. Here we reveal for the first time by using density functional theory calculations that framework hexa-coordinated Ti (TiO_6_) species of mesoporous titanium silicates are the most active sites for ODS and lead to a lower-energy pathway of ODS. A novel method to achieve highly accessible and homogeneously distributed framework TiO_6_ active single sites at the mesoporous surface has been developed. Such surface framework TiO_6_ species exhibit an exceptional ODS performance. A removal of 920 ppm of benzothiophene is achieved at 60°C in 60 min, which is 1.67 times that of the best catalyst reported so far. For bulky molecules such as 4,6-dimethyldibenzothiophene (DMDBT), it takes only 3 min to remove 500 ppm of DMDBT at 60°C with our catalyst, which is five times faster than that with the current best catalyst. Such a catalyst can be easily upscaled and could be used for concrete industrial application in the ODS of bulky organosulfur compounds with minimized energy consumption and high reaction efficiency.

## Teaser

Titanium silicate catalysts with highly accessible and homogeneously distributed framework TiO_6_ active single sites at the mesoporous surface were obtained by engineering electrostatic interface, exhibiting unprecedented deep oxidative desulfurization performance.

## INTRODUCTION

Sulfur compounds in fossil fuels not only provoke the corrosion of combustion engines, but also severely damage human health and the environment [1–3]. Gasoline obtained from fluid catalytic cracking contains thiophenic sulfurs with a content of 500–1600 ppm [4–6]. Efficient deep desulfurization techniques are urgently needed to reduce the sulfur content in fuels to <10 ppm to meet the stringent environmental regulations [7,8].

The hydrodesulfurization (HDS) technique is able to remove some sulfur compounds under harsh reaction conditions [9,10] but shows poor performance for thiophenic sulfurs such as benzothiophene (BT), dibenzothiophene (DBT) and 4,6-dimethyldibenzothiophene (DMDBT). The high-density electrons on the sulfur atom undergo a delocalization toward aromatic rings, which creates extra stability and renders HDS inefficient for breaking the C–S bonds of thiophenic sulfurs and thus severely limits deep desulfurization [11,12]. The oxidative desulfurization (ODS) technique has emerged as a promising way to remove thiophenic sulfurs to achieve deep desulfurization [8,13,14]. Titanium silicate materials such as titanium silicalite-1 (TS-1) zeolite with framework tetra-coordinated Ti (TiO_4_) sites show good ability to catalyse ODS. However, the activity of the conventional TS-1 zeolites for ODS is barely satisfying [15–17]. Framework TiO_4_ is linked with four Si atoms via bridged O atoms, which is a relatively closed structure [18–20], while thiophenic sulfurs such as BT, DBT and DMDBT are bulky molecules that are hard to access with such closed sites located in the micropores of the zeolite framework, thus leading to a low activity. On the other hand, conventional TS-1 zeolites have narrow micropores (<0.56 nm), which severely restrains the diffusion and transport of bulky thiophenic sulfur molecules, resulting thus in poor performance [15–17,21]. Titanium silicate materials with highly accessible and open Ti active sites are highly demanding to achieve deep ODS.

Apart from the framework TiO_4_ active sites, it has been reported that there is another type of Ti site—framework hexa-coordinated Ti (TiO_6_) with two water and two hydroxyl ligands, linked with only two Si atoms via bridged O atoms [18–20]. Such framework TiO_6_ species were revealed to have even better activity than framework TiO_4_ in the oxidation of some alkenes and thiophenic sulfurs. Guo *et al.* were the first to identify framework TiO_6_ in TS-1 zeolites by using *in situ* ultraviolet Raman (UV–Raman) spectroscopy and found that framework TiO_6_ is more active than framework TiO_4_ in the epoxidation of propene [18]. For 1-hexene, mononuclear framework TiO_6_ species were demonstrated to achieve higher epoxidation activity than the conventional framework TiO_4_ species [20]. Moreover, framework TiO_6_ in titanium silicate materials was found to significantly outperform framework TiO_4_ not only for the epoxidation of cyclohexene, but also for the ODS of DBT [21]. The superior activity of the framework TiO_6_ compared with the framework TiO_4_ is partly ascribed to its more open structure, which offers much better interaction with bulky reactant molecules. Unfortunately, this type of framework TiO_6_ active site is still neglected as the precise control of uniform framework TiO_6_ sites in titanium silicates remains highly challenging.

For the first time, we reveal by using density functional theory calculations that framework TiO_6_ sites orient a much lower-energy pathway of the ODS process due to a much lower adsorption energy of the oxidant (–78.7 kJ/mol) than that on the framework TiO_4_ sites (–22.2 kJ/mol). Following this guidance, a synthesis method to create highly accessible and uniformly distributed framework TiO_6_ single sites on the mesopore surface by engineering the electrostatic interface between negatively charged Ti species and positively charged surfactant molecules is developed. The obtained mesoporous titanium silicate material exhibits extraordinary ODS performance, achieving complete removal of BT, DBT and DMDBT from model fuels. Under a reaction at 60°C for 60 min, our catalyst can reach a BT conversion of 920 ppm that is 1.67 times that of the best catalyst reported so far. Even for a bulky molecule such as DMDBT, it takes only 3 min to achieve a conversion of 500 ppm at 60°C with our catalyst, which is five times faster than that with the current best catalyst. Our catalyst also possesses great reusability with highly stable activity over five reaction cycles. Our synthesis method is economically feasible and easily up-scalable for large-scale production toward their use in the industrial deep ODS of bulky sulfur compounds.

## RESULTS AND DISCUSSION

**Figure 1.**
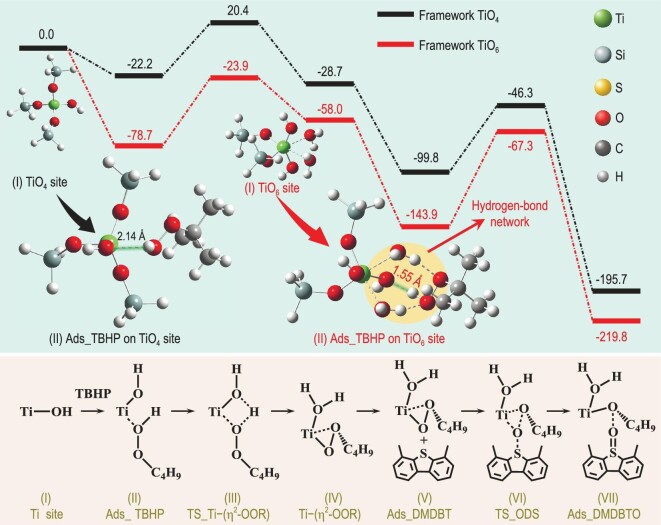
Reaction pathway and the corresponding calculated energies for the ODS of DMDBT over a framework TiO_4_ site and a framework TiO_6_ site, including 10 steps. (Step I) Ti site: the framework TiO_4_ site or framework TiO_6_ site; (Step II) Ads_TBHP: the adsorption of an oxidation TBHP; (Step III) TS_Ti–(*η*^2^–OOR): the transition state of an active intermediate; (Step IV) Ti–(*η*^2^–OOR): the active intermediate; (Step V) Ads_DMDBT: the adsorption of DMDBT molecules; (Step VI) TS_ODS: the transition state of the ODS reaction; (Step VII) Ads_DMDBTO: the formation of oxide product DMDBTO. Steps VIII–X and the corresponding calculated energies are shown in Fig. S1. R represents –C(CH_3_)_3_. All energies are given in kJ/mol. The insets of the energy plot: calculated structures showing the adsorption of an oxidation TBHP over a framework TiO_4_ site or a framework TiO_6_ site. The orange area shows a hydrogen-bond network in (Step II) Ads_TBHP over a framework TiO_6_ site. Other calculated structures of Steps III–X are shown in Figs S2 and S3.

Theoretical calculations based on density functional theory were first conducted to investigate the mechanism of the ODS reaction over framework TiO_6_ and TiO_4_ sites. DMDBT has been selected as a model reactant molecule of ODS while tert-butyl hydroperoxide (TBHP, (CH_3_)_3_COOH) is used as the oxidant because it is oil-soluble, highly oxidative, thermostable (>80°C) and used for industrial continuous processes [22–24]. As shown in Fig. 1 and Figs S1–S3, ODS over both the framework TiO_4_ and framework TiO_6_ sites follow a typical 10-steps pathway [25,26]. Ti site (Step I) adsorbs a TBHP to form Ads_TBHP (Step II). This structure evolves into a transition state (Step III, TS_Ti–(*η*^2^–OOR), R represents –C(CH_3_)_3_) and reaches the active intermediate (Step IV, Ti–(*η*^2^–OOR)). Further adsorption of a DMDBT molecule leads to the formation of Ads_DMDBT (Step V), which evolves into a transition state for the ODS reaction (Step VI, TS_ODS) and produces an adsorbed DMDBT oxide (DMDBTO) (Step VII, Ads_DMDBTO). Subsequently, DMDBTO desorbs (Step VIII, Ti–OR) and transforms into a transition state (Step IX, TS_ROH). Finally, the Ti site is recovered (Step X, Ti site recovery) by using the desorption of ROH. It can be obviously observed that the ODS reaction on the framework TiO_6_ site occurs via a much lower-energy pathway than that on the framework TiO_4_ site throughout the whole reaction (Fig. 1, Fig. S1 and Table S1), which is mainly contributed by the much lower adsorption energy of TBHP (Steps I and II) over the framework TiO_6_ site (–78.7 kJ/mol) than that over the framework TiO_4_ site (–22.2 kJ/mol). The calculated structures of Step II (Ads_TBHP) in Fig. 1 indicate that the lower adsorption energy of TBHP over the framework TiO_6_ site is ascribed to the strong interaction between the –OOH group of (CH_3_)_3_COOH and two coordinated H_2_O of the framework TiO_6_ site provided by a hydrogen-bond network. This is further evidenced by using a much shorter bond length of TiO···HOOR over the framework TiO_6_ site (1.55 Å) than that over the framework TiO_4_ site (2.14 Å). All the above results clearly suggest that the framework TiO_6_ sites are the most active sites for catalytic ODS.

**Figure 2.**
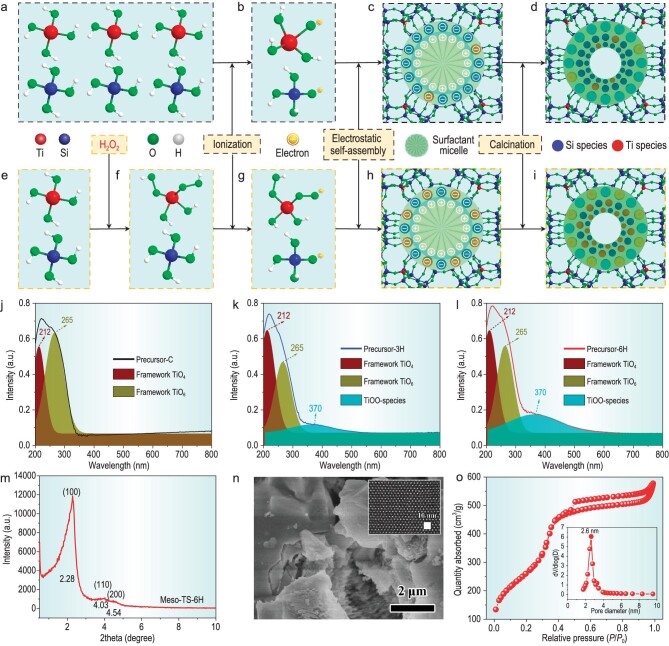
Schematic presentation of a synthesis method to engineer the electrostatic interface to construct surface framework TiO_6_ single sites. The traditional synthesis process of Meso-TS-C: (a) hydrolysis products, (b) ionization products, (c) product from a conventional self-assembly process, (d) material with a small amount of framework TiO_4_ on the mesopore surface. Proposed preparation process of Meso-TS-3H and 6H: (e) hydrolysis products, (f) TiOOH species formed by adding H_2_O_2_, (g) TiOO^–^ species ionized from TiOOH, (h) product of an accelerated electrostatic self-assembly process, (i) material with abundant framework TiO_6_ on the mesopore surface. UV–vis spectra of (j) Precursor-C, (k) Precursor-3H and (l) Precursor-6H before a calcination treatment. Characterizations of Meso-TS-6H synthesized by engineering the electrostatic interface: (m) SAXD pattern, (n) SEM image and (the inset) high-resolution TEM image showing highly ordered mesopores, (o) N_2_ adsorption–desorption isotherm and (the inset) pore-size distribution.

Guided by using theoretical calculation results, a synthesis method to create highly accessible and uniform framework TiO_6_ single sites on a mesopore surface to avoid the drawbacks of microporous TS-1 zeolites in terms of accessibility to active sites and of unsatisfactory activity of TiO_4_ sites has been developed by engineering the electrostatic interface. It is reported that a typical electrostatic interface between positively charged ionic surfactant molecules (cetyltrimethylammonium bromide) and negatively charged titanium silicate species exists during the synthesis of mesoporous titanium silicates (denoted as Meso-TS-C, C represents cetyltrimethylammonium bromide) [27,28]. For the preparation of Meso-TS-C, the hydrolysis product containing SiO–H and TiO–H tends to ionize much more into SiO^–^ species rather than TiO^–^ species, since the electronegativity of Si (1.90) is higher than that of Ti (1.54) (Fig. 2a and b). Therefore, negatively charged Si species would dominate the occupation of the electrostatic interface (Fig. 2c), leading to a few accessible TiO_4_ sites on the mesopore surface of Meso-TS-C (Fig. 2d), which would severely restrict the catalytic performance. Herein, H_2_O_2_ is innovatively introduced into hydrolysis products to *in situ* transform TiOH into TiOOH (Fig. 2e and f). This precisely designed method is on the basis that the first ionization constant of H_2_O_2_ (K_1_ = 1.55 × 10^–12^) is 155 times higher than that of H_2_O (K_1_ = 1.00 × 10^–14^), which indicates that TiOO–H will possess a more highly enhanced ionization ability than TiO–H to produce considerable TiOO^–^ in a basic solution (Fig. 2f and g). Such TiOO^–^ species would occupy the electrostatic interface by an electrostatic interaction (Fig. 2h) and subsequently turn into highly accessible Ti sites on the mesopore surface after final calcination treatment (Fig. 2i). According to the introduction amount of H_2_O_2_ solution (30 wt%) (designed to be three or six times as much as the Ti source added), the products are denoted as Meso-TS-3H or 6H. As shown in Fig. S4, the mother liquid of Meso-TS-C exhibits a white color that transforms into pale yellow (Meso-TS-3H) and yellow (Meso-TS-6H) after adding the H_2_O_2_ solution. The color evolution of the mother liquid is ascribed to the transformation of TiOH into TiOOH [29–33]. During treatment at 60°C (Fig. S4), the mother liquid of Meso-TS-C takes 12 h to complete the precipitation process, which is much longer than Meso-TS-3H (1 h) and Meso-TS-6H (30 min). The obviously faster precipitation process suggests that TiOOH is able to ionize into abundant negatively charged Ti species, leading to a faster electrostatic self-assembly process.

In order to gain further insight into the negatively charged Ti species, after a complete electrostatic self-assembly process at 100°C for 2 days, the intermediate products were separated by washing and drying at 60°C but without the final calcination treatment (denoted as Precursor-C, Precursor-3H or 6H) to capture the state of the Ti during the electrostatic interaction with surfactants. The ultraviolet-visible diffuse reflectance (UV–vis) spectra of Precursor-C, Precursor-3H and 6H all show absorption peaks featuring at 212 and 265 nm attributed to framework tetra-coordinated Ti (TiO_4_) and framework TiO_6_, respectively (Fig. 2j–l). These results indicate that Ti was incorporated into the framework during the self-assembly process. However, these framework Ti sites are normally distributed in the pore wall of mesoporous titanium silicates, and are thus inaccessible to reactant molecules. Importantly, one absorption peak at 375 nm corresponding to a charge transfer from the peroxide moiety to the Ti center [29–33] is only observed in Precursor-3H and 6H, along with the powder color change from white to pale yellow and yellow (Fig. 2k and l, and Fig. S5). These results confirm the negatively charged Ti species to be peroxide moiety interacting with framework Ti ions (TiOO^–^) that are ionized from TiOO–H. These TiOO^–^ species are inferred to be located at the interface via electrostatic interaction with positively charged surfactants and the resultant Ti sites will be highly accessible. All the above results clearly demonstrate that, by introducing H_2_O_2_ into the hydrolysis product, the resultant TiOOH possesses an enhanced ionization ability to produce a large amount of TiOO^–^, which promotes the electrostatic interaction, leading to an accelerated self-assembly process and an electrostatic interface full of Ti species. Our synthesis method to engineer the electrostatic interface is conducive to the formation of considerable Ti sites on the surface. Such surface Ti sites possess excellent accessibility to reactants and might exhibit excellent catalytic performance.

After the final calcination treatment of the intermediate products under 550°C, it can be seen that the small-angle X-ray diffraction (SAXD) pattern of Meso-TS-C (Fig. S6a) shows one diffraction peak at 2.28° in accordance with the (100) face of the p6 mm structure of the ordered mesoporous framework [27,34], evidencing the successful preparation of mesoporous titanium silicate material. As the introduction amount of H_2_O_2_ increases, the intensity of this diffraction peak increases and two other diffraction peaks at 4.03° and 4.54° attributed to the (110) and (200) faces of the mesoporous framework [27,34], respectively, appear in Meso-TS-3H and 6H (Fig. S7a and Fig. 2m), indicating the formation of highly ordered hexagonally organized mesopores from a promoted electrostatic self-assembly process. The scanning electron microscopy (SEM) image shows that Meso-TS-C (Fig. S6b) possesses a multilayered morphology with a considerable number of small particles on the surface. For Meso-TS-3H and 6H (Fig. S7b and Fig. 2n), an intact multilayered morphology is observed, indicating that an enhanced electrostatic self-assembly process is conducive to the formation of ordered mesopores. N_2_ adsorption–desorption results of Meso-TS-C (Fig. S6c) show a typical type IV isotherm with a hysteresis loop at a relative pressure from 0.50 to 0.99, confirming the existence of mesopores. The mesopore diameter, Brunauer-Emmett-Teller (BET) surface area and mesopore volume are determined to be 2.7 nm, 827 cm^2^/g and 0.82 cm^3^/g, respectively (Fig. S6d and Table S2). Meso-TS-3H and 6H (Fig. S7c and Fig. 2o) also possess a type IV isotherm with an obvious hysteresis loop at a relative pressure from 0.50 to 0.99. The mesopore diameter is almost the same (∼2.6 nm) but the BET surface area increases to 1269 and 1201 cm^2^/g, and the mesopore volume is as high as 0.79 and 0.89 cm^3^/g, respectively (Fig. S7d, Fig. 2o and Table S2). The introduction of H_2_O_2_ into the mother liquid results in products with high quality embodying in highly ordered mesopores, large surface areas and high pore volumes. The transmission electron microscopy (TEM) image (Fig. S8a) shows one layer of Meso-TS-6H, which is consistent with the SEM result. The high-resolution TEM images of Fig. S8b–d and the inset of Fig. 2n evidence the p6 mm structure of the ordered mesoporous framework. The high-angle annular dark field (HAADF)-scanning transmission electron microscopy (STEM)-energy dispersive X-ray mappings (Fig. S9) depict the homogeneous distribution of Ti without any other phase observed, indicating a full condensation of titanium silicates in Meso-TS-6H by an enhanced electrostatic self-assembly process.

**Figure 3.**
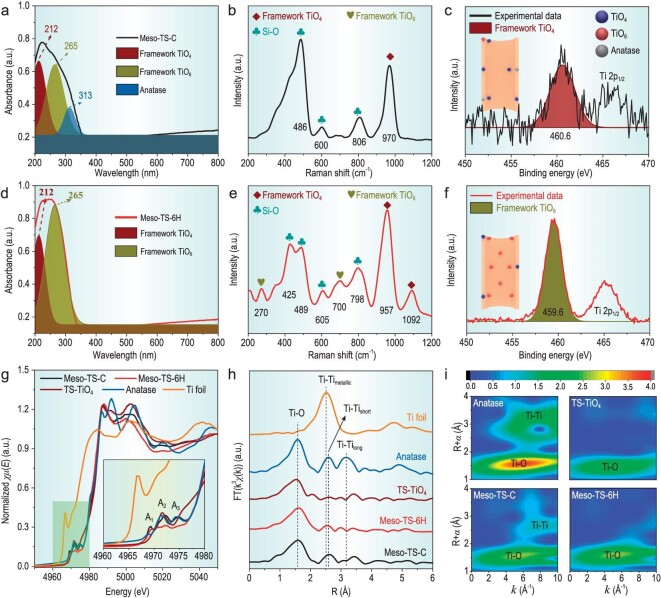
Characterizations of the state of Ti species. (a and d) UV–vis spectra, (b and e) Raman spectra, and (c and f) XPS spectra of Ti 2p_3/2_ of (a–c) Meso-TS-C and (d–f) Meso-TS-6H; the signal at ∼466 eV in the XPS spectra of (c and f) is assigned to Ti 2p_1/2_. (g) Ti K-edge XANES spectra, (h) Fourier-transform (FT) EXAFS spectra and (i) wavelet transform for the *k*^3^-weighted EXAFS of Meso-TS-C, Meso-TS-6H and reference samples.

The state of the Ti in the materials was investigated by using UV–vis, UV–Raman and X-ray photoelectron spectroscopy (XPS) techniques. The UV–vis technique can detect the distribution of Ti in the whole materials. UV–Raman spectroscopy only reveals the Ti state near the surface and the XPS technique is utilized to further confirm the surface composition. Three absorption peaks at 212, 265 and 313 nm that are ascribed to framework TiO_4_, framework TiO_6_ and anatase [18–20,35], respectively, can be observed in the UV–vis spectrum of Meso-TS-C (Fig. 3a). The Raman spectrum of Meso-TS-C (Fig. 3b) shows resonance signals at 486, 600 and 806 cm^–1^ belonging to Si–O as well as a signal at 970 cm^–1^ ascribed to framework TiO_4_ [35,36]. It is reported that the signals centering at ∼460.6, ∼459.6 and ∼458.6 eV in the XPS results are assigned to framework TiO_4_, framework TiO_6_ and anatase, respectively [19,37]. The XPS spectrum of Ti 2p_3/2_ of Meso-TS-C (Fig. 3c) confirms the sole existence of a small amount of framework TiO_4_ at the surface by showing only one peak at 460.6 eV [37]. These results demonstrate that Meso-TS-C obtained by using a conventional synthesis method possesses framework TiO_4_, framework TiO_6_ and anatase in the pore walls, while sole-framework TiO_4_ is found on the pore surface with a very low surface Ti content of 0.32% (atomic content determined by using XPS). The complex Ti state and insufficient Ti sites on the mesopore surface of the mesoporous titanium silicates have been crucial problems for a long time. By engineering the electrostatic interface, UV–vis spectra of Meso-TS-3H and 6H (Fig. S10a and Fig. 3d) exhibit only two absorption peaks featuring at 212 and 265 nm attributed to framework TiO_4_ and framework TiO_6_, respectively [18–20,35]. The intensity of the band at 265 nm increases from Meso-TS-3H to Meso-TS-6H and this band in Meso-TS-6H becomes dominant. They also possess Raman resonance signals at ∼425, ∼486, ∼600 and ∼806 cm^–1^ belonging to Si–O and signals at ∼970 and ∼1092 cm^–1^ ascribed to framework TiO_4_ (Fig. S10b and Fig. 3e) [35,36]. Notably, one Raman resonance signal at ∼700 cm^–1^ [18–20] attributed to framework TiO_6_ appears only in the Raman spectra of Meso-TS-3H and 6H, indicating the formation of framework TiO_6_ near the surface. The Ti state on the mesopore surface of Meso-TS-3H and 6H is further confirmed to be only framework TiO_6_ as demonstrated by one peak at ∼459.6 eV in the XPS spectra (Fig. S10c and Fig. 3f) [37]. The Ti content on the surface is significantly promoted to be 1.43% and 1.47% for Meso-TS-3H and 6H, respectively. All the above results demonstrate that engineering the electrostatic interface promotes the occupation of TiOO^–^ species on the electrostatic interface and mitigates the formation of extra-framework anatase. Such TiOO^–^ species transform into abundant framework TiO_6_ sites on the pore surface after the final calcination treatment.

To further investigate the coordination environment of Ti species, X-ray absorption near-edge structures (XANES) and extended X-ray absorption fine structures (EXAFS) were conducted on the Meso-TS materials and reference samples (anatase, Ti foil and a TS-1 zeolite containing only TiO_4_ (TS–TiO_4_)). The XANES spectra can reflect the symmetry of the Ti coordination structure in the range of pre-edge peaks [20,38,39]. Apart from Ti foil with high symmetry of the Ti coordination structure, the highest peak can be observed in the TS–TiO_4_ at A_2_, indicating that TiO_4_ is a highly symmetric structure (Fig. 3g and the enlarged area). As for the anatase, three peaks arise at A_1_, A_2_ and A_3_. These three peaks can also be observed in Meso-TS-C due to the existence of some anatase phase. Regarding Meso-TS-6H, only one peak at A_1_ can be observed, indicating the absence of anatase phase. Moreover, the intensity of this peak is significantly reduced, which demonstrates a much less symmetrical coordination structure of Ti in Meso-TS-6H due to the existence of TiO_6_ that has proven to be a distorted structure [20]. The Fourier-transformed extended X-ray absorption fine structure (FT-EXAFS) spectra (Fig. 3h) of the Ti foil and anatase show peaks attributed to Ti–O and Ti–Ti (Ti–Ti_metallic_, Ti–Ti_short_ and Ti–Ti_long_) bonds [20,38,39]. Notably, Meso-TS-6H and TS–TiO_4_ show only one major peak of Ti–O with no obvious Ti–Ti bond, suggesting that both TiO_6_ and TiO_4_ species are single sites, confirming the successful synthesis of framework TiO_6_ single sites on the mesopore surface. As for Meso-TS-C, one minor peak of Ti–Ti_short_ can be observed due to the existence of some anatase phase. The fitting results of the Ti–O bond in the FT-EXAFS spectra (Fig. S11 and Table S3) reveal that the coordination number of the Ti–O path in Meso-TS-6H is 4.1 times larger than that of TS–TiO_4_ (3.3), evidencing that framework TiO_6_ single sites are highly coordinated. Wavelet transform (WT) analysis was used to investigate the EXAFS oscillations to provide resolutions in radial distance and in k-space [20,38,39]. As shown in Fig. 3i, all WT analyses display a Ti–O shell featuring at 1–2 Å while the anatase and Meso-TS-C show an additional WT maximum at 2–4 Å, which is ascribed to the Ti–Ti shell. It is worthy to note that no Ti–Ti shell can be observed in Meso-TS-6H or TS-TiO_4_, further verifying that TiO_6_ species are single sites. Similarly, the XANES and EXAFS results of Meso-TS-3H (Fig. S12 and Table S3) also indicate that TiO_6_ species in Meso-TS-3H are single sites. All these results clearly confirm that the obtained framework TiO_6_ sites in the mesopore surface are a highly coordinated, single-site structure.

To evidence the importance of the electrostatic interface in the formation of framework TiO_6_ single sites, a mesoporous titanium silicate material assembled by using nonionic surfactants was synthesized according to the same strategy and labeled as Meso-TS-N (N represents nonionic surfactants) (Fig. S13). Meso-TS-N shows a sole anatase phase on the surface (Fig. S14), which indicates that materials synthesized with nonionic surfactant molecules cannot form an electrostatic interface, further evidencing the importance of engineering the electrostatic interface for the precise control of surface composition. Our mesoporous materials with highly accessible and uniformly distributed framework TiO_6_ single sites of mesopore surface may lead to extraordinary catalytic performance.

**Figure 4.**
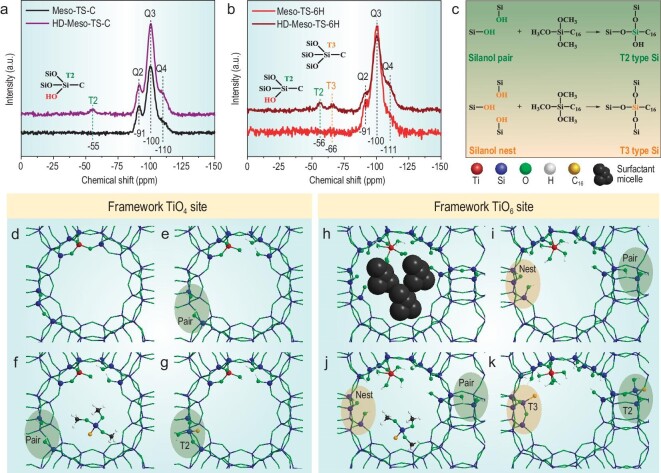
^29^Si CP–MAS NMR spectra of (a) conventional Meso-TS-C and (b) Meso-TS-6H before and after silylation with hexadecyltrimethoxysilane ((CH_3_O)_3_–Si–C_16_) with a –Si–C_16_ fragment. (c) Schematic presentation of the silylation reaction between silanols in titanium silicate materials and (CH_3_O)_3_–Si–C_16_: reaction with a silanol pair to produce a T2 type of Si atom and reaction with a silanol nest to produce a T3 type of Si atom. Schematic presentation of the mechanism of the open structure of framework TiO_6_ compared with framework TiO_4_: (d) a close framework TiO_4_ site, (e) silanol types in Meso-TS-C, (f) spatial relationship between silanol and (CH_3_O)_3_–Si–C_16_ in Meso-TS-C during silylation. (g) T2-type Si atoms form in Meso-TS-C after silylation; (h) an electrostatic interface between negatively charged TiOO^–^ and positively charged cetyltrimethylammonium; (i) a framework TiO_6_ site with an open structure after calcination and silanol types in Meso-TS-6H; (j) spatial relationship between silanol and (CH_3_O)_3_–Si–C_16_ in Meso-TS-6H during silylation; (k) the formation of T2- and T3-type Si atoms in Meso-TS-6H after silylation.

In order to investigate the open structure of the surface framework TiO_6_ single sites, silylation of bulky –Si–C_16_ fragments that possess similar structure and molecular size to cetyltrimethylammonium ion (CTA^+^) onto the surface of samples was utilized to recur the spatial structure around Ti sites after removing the CTA^+^ surfactants. ^29^Si cross polarization (CP)–magic angle spinning (MAS) nuclear magnetic resonance (NMR) spectra of Meso-TS-C and Meso-TS-6H (Fig. 4a and b) show resonance peaks at around –91, –100 and –113 ppm, which are attributed to Q2 ((HO)_2_–Si–(OSi)_2_), Q3 (HO–Si–(OSi)_3_) and Q4 (Si–(OSi)_4_) types of Si, respectively [40,41]. After silylation treatment with hexadecyltrimethoxysilane (HDTMOS, (CH_3_O)_3_–Si–C_16_), the obtained HD-Meso-TS-C (HD represents hexadecyl) and HD-Meso-TS-6H both exhibit the transformation of Q2 and Q3 into Q4 types of Si (Fig. 4a and b), indicating the successful condensation between surface Si–OH and –Si–C_16_ fragments to produce (Si–O)*_x_*–Si–C_16_ (*x* = 1–3) structures. These structures vary according to the grafting sites (Si–OH) on the surface of materials. As shown in Fig. 4c, grafting on a silanol pair produces a T2 type of Si ((Si–O)_2_–Si–C_16_) while grafting on a silanol nest yields a T3 type of Si ((Si–O)_3_–Si–C_16_) [42,43]. The ^29^Si CP–MAS NMR spectrum of HD-Meso-TS-C (Fig. 4a) shows a resonance peak at –55 ppm ascribed to a T2 type of Si, indicating the existence of silanol pairs. Notably, HD-Meso-TS-6H (Fig. 4b) exhibits an additional resonance peak at –66 ppm attributed to a T3 type of Si [40], indicating that there exist unique silanol nests accessible for silylation with bulky –Si–C_16_ fragments in Meso-TS-6H. These accessible silanol nests can be reasonably attributed to the exclusive surface framework TiO_6_ single sites in Meso-TS-6H. Silanol pairs are commonly observed in Si–O–Si frameworks due to atom vacancies during synthesis [43], which contributes to the formation of a T2 type of Si in HD-Meso-TS-C and HD-Meso-TS-6H after silylation (green area in Fig. 4d–k). In addition to silanol pairs, Si atoms adjacent to TiO_4_ sites tend to form Si vacancies to produce silanol nests (Fig. 4d and e) for catalytic ability [25,35]. However, these silanol nests cannot accommodate the incorporation of a bulky –Si–C_16_ fragment (Fig. 4f and g) due to the closed structure of the framework TiO_4_ sites, as demonstrated by no T3 type of Si in HD-Meso-TS-C (Fig. 4a). As for the surface of Meso-TS-6H with sole-framework TiO_6_ single sites, Ti sites link with only two Si atoms, providing more Si vacancies and silanol nests around Ti sites after removing the surfactants (orange area in Fig. 4h and i). This open structure endows silanol nests with the unique ability to accommodate a bulky –Si–C_16_ fragment (Fig. 4j and k), producing HD-Meso-TS-6H with a T3 type of Si (Fig. 4b). All the above results clearly confirm the open structure of the surface framework TiO_6_ single sites. Such highly accessible framework TiO_6_ single sites with an open structure may possess unimpeded interaction with bulky thiophenic sulfurs, leading to highly efficient ODS performance.

**Figure 5.**
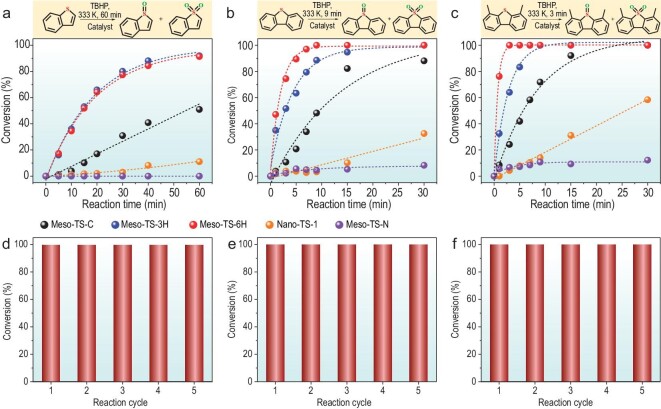
ODS performance with different catalysts. (a) BT conversion, (b) DBT conversion and (c) DMDBT conversion as a function of the reaction time. Reusability of Meso-TS-6H for the ODS of (d) BT at 70°C for 60 min, (e) DBT at 60°C for 30 min and (f) DMDBT at 60°C for 30 min for five reaction cycles.

The superiority of such a mesopore surface with highly accessible framework TiO_6_ single sites with an open structure is demonstrated in the catalytic ODS using BT, DBT and DMDBT as probe molecules. The above-mentioned Meso-TS-N and the current widely used ODS catalyst, a nanosized microporous TS-1 zeolite (Nano-TS-1, Figs S15 and S16), are used as references. Herein, the model fuel of BT, DBT and DMDBT contains 1000, 1000 and 500 ppm of thiophenic sulfurs, respectively. As expected, Meso-TS-N with only anatase on the mesopore surface is almost inactive for all ODS reactions (Fig. 5a–c). Notably, the best ODS performance of BT, DBT and DMDBT was obtained over our Meso-TS-3H and 6H catalysts. As shown in Fig. 5a, Meso-TS-3H and Meso-TS-6H achieved a BT conversion of 92% and 91% in 60 min at 60°C, respectively, both outperforming Meso-TS-C (51%) and Nano-TS-1 (11%). A removal of 920 ppm of BT was achieved on our Meso-TS-3H catalyst in 60 min at 60°C, which is 1.67 times that of the best catalyst (550 ppm) reported so far (Table S4) [13]. It is worth noting that the BT conversion with our catalysts can be promoted to 100% by elevating the reaction temperature to 70°C (see temperature modulation results in Fig. S17), achieving deep desulfurization (S < 10 ppm). Regarding the ODS of DBT (Fig. 5b), Meso-TS-3H and Meso-TS-6H totally removed DBT at 60°C in 30 and 9 min, respectively. Meso-TS-C and Nano-TS-1 only achieved a DBT conversion of 48% and 3% in 9 min, respectively. Notably, our Meso-TS-6H catalyst removed 740 ppm of DBT in 3 min, which is 1.85 times that of the current best catalyst by far (400 ppm, Table S4) [44]. For the ODS of the much bulkier DMDBT molecule at 60°C, even better ODS performance can be obtained on Meso-TS-3H and Meso-TS-6H. They can completely remove DMDBT in only 7 and 3 min, respectively, which is highly superior to Meso-TS-C (24% in 3 min) and Nano-TS-1 (4% in 3 min) (Fig. 5c). Moreover, the Meso-TS-6H catalyst removed 500 ppm of DMDBT in 3 min, which is five times faster than the current best catalyst (15 min, Table S4) [13]. The low catalytic activity of Meso-TS-C in the ODS reaction is due to the low activity of TiO_4_ species, while the poor performance of the nanosized microporous TS-1 zeolite is attributed to the small pores of the zeolite, which hinder the diffusion of bulky organosulfur molecules in the micropores. All the above results demonstrate that Meso-TS-3H and 6H are highly superior ODS catalysts for achieving deep desulfurization compared with the current best catalysts (Table S4), exhibiting the best ODS performance reported so far. Such extraordinary performance can be attributed to the highly accessible, highly active framework TiO_6_ single sites on the mesopore surface, and the superior diffusion and transport ability provided by the highly ordered mesopores. Meso-TS-6H outperforms Meso-TS-3H due to its more ordered mesopores (X-ray diffraction (XRD) patterns in Fig. 2m and Fig. S7a) and higher mesopore volume (0.89 versus 0.79 cm^3^/g in Table S2), suggesting the importance of highly ordered mesopores in the diffusion and transport of reactant molecules. The slower removal rate of BT than DBT and DMDBT is because the electrons of S in BT can only delocalize toward one aromatic ring, rendering BT harder to oxidize than DBT and DMDBT with two aromatic rings, as evidenced by a much higher apparent activation energy of 104 kJ/mol than that of DBT (71 kJ/mol) and DMDBT (71 kJ/mol), as shown in Fig. S17. Importantly, our Meso-TS-6H retains 100% conversion of BT, DBT and DMDBT after five reaction cycles and multiple calcination treatments at 550°C, demonstrating the excellent thermal stability of framework TiO_6_ and mesopores (Fig. 5d–f). Meso-TS-6H shows no obvious activity loss after seven reaction cycles and retains a DMDBT conversion of 75% after 10 reaction cycles (Fig. S18). As for Meso-TS-C, the activity keeps declining during 10 reaction cycles and reaches a DMDBT conversion of only 8% after the final reaction cycle (Fig. S18). The obviously faster deactivation of Meso-TS-C is ascribed to the much more severe structural collapse and loss of Ti sites in comparison with Meso-TS-6H (Figs S19 and S20), demonstrating that the highly improved electrostatic self-assembly process is conducive to promoting the stability of mesoporous materials. It is extremely difficult for the current industrial hydrodesulfurization (HDS) process to remove thiophenic sulfurs such as BT, DBT and DMDBT [8]. ODS with our catalyst can easily remove DBT and DMDBT from model fuels. All the above results strongly indicate that our materials possessing surface framework TiO_6_ single sites with an open structure are the best ODS catalysts reported so far, solving the current industrial desulfurization problem and thus achieving deep desulfurization. Our catalyst with framework TiO_6_ sites are also found to be able to effectively catalyse the epoxidation reaction of cyclohexene and styrene, exhibiting good universality (Fig. S21). This novel catalyst is envisioned to be utilized to catalyse extensive reactions for the efficient production of chemicals and commodities.

## CONCLUSION

Theoretical calculations were conducted for the first time and revealed that framework TiO_6_ is much more active than the conventional framework TiO_4_ for catalytic ODS because of a lower adsorption energy of the oxidation (reduced from –22.2 to –78.7 kJ/mol). A synthesis method to achieve highly accessible and highly active framework TiO_6_ single sites on the mesopore surface was developed by engineering the electrostatic interface. Such materials exhibited the best ODS activity reported so far for a series of thiophenic sulfurs. For example, our catalyst completely removed 500 ppm of DMDBT from model fuel at 60°C in 3 min, which is five times faster than the current best catalyst (15 min). The presented highly active mesoporous titanium silicate could be used for concrete industrial application in the ODS of bulky organosulfur compounds.

## METHODS

### Preparation of Meso-TS-3H and 6H

Typically, 5.49 g of diethylamine (Aladdin, AR) was dissolved into 108 g of diluted water, followed by adding 2.73 g of cetyltrimethylammonium bromide (Aladdin, AR). The solution was vigorously stirred for 30 min. Hereafter, 0.68 g of tetrabutyl titanate (TBT, Aladdin, AR) was dissolved into 10.42 g of tetraethyl orthosilicate (Aladdin, AR) to form a homogeneous mixture. The mixture was dropwise added into the above solution. After vigorous stirring for 4 h, a H_2_O_2_ solution with a mass of three or six times as much as the TBT added was introduced and the stirring was maintained for another 30 min. The hydrothermal process was conducted in a Teflon lining sealed by a stainless-steel autoclave at 100°C for 2 days. Finally, the obtained powder was collected by filtering, washed with deionized water as well as alcohol and dried at 120°C overnight. The product was obtained after calcination at 550°C (ramp rate = 5°C/min) in air for 6 h in a muffle furnace. According to the mass ratio of the H_2_O_2_ solution to the TBT, the obtained mesoporous titanium silicates were denoted as Meso-TS-3H or Meso-TS-6H.

### Catalyst characterizations

Powder XRD patterns were recorded on a D8 ADVANCE with Cu Kɑ radiation (λ = 1.5413 Å). SAXD were recorded on a Rigaku Ultimate IV. The SEM images were collected using a Hitachi S-4800 scanning electron microscope. The TEM and high-resolution TEM (HR-TEM) images were investigated by using a JEOL JEM-2100F HRTEM. N_2_ adsorption–desorption isotherms were recorded using a Micromeritics ASAP 3020 gas sorptiometer after the samples were degassed at 300°C under a vacuum for 8 h. Lambda 750 S equipment was applied to obtain the UV–vis diffuse reflectance spectrum with BaSO_4_ as the reference sample. The UV–Raman spectrum was recorded via a Horiba Evolution spectrometer using the 325-nm line of a He–Cd laser as the excitation source. XPS was performed on a Thermo Scientific K-Alpha+ (Thermo Fisher) spectrometer equipped with a mono Al–Kα X-ray source (excitation energy = 1486.6 eV). Inductively coupled plasma optical emission spectrometer (ICP–OES, Agilent 730) was used to determine the element fraction of the samples. The NMR spectra were recorded at room temperature using a JEOL ECZ-R spectrometer operating at 14.1 T (^29^Si frequency = 119.2 MHz) equipped with a 3.2-mm AUTOMAS probe. The X-ray absorption fine structure and EXAFS of the Ti K-edge were performed at the BL11B beamlines at the Shanghai Synchrotron Radiation Facility. Calculations were carried out using the Gaussian 09 program suite based on density functional theory methods. The catalytic performance was evaluated in the ODS of BT, DBT and DMDBT. More synthesis and characterization details can be found in the Supporting Information.

## Supplementary Material

nwae085_Supplemental_File
