# Supplementary material for: Engineering surface framework TiO6 single sites for unprecedented deep oxidative desulfurization
Source: Natl Sci Rev. 2024 Mar 6;11(5):nwae085. doi: 10.1093/nsr/nwae085 (PMC10989657; doi:10.1093/nsr/nwae085)
Supplement: nwae085_Supplemental_File [file nwae085_supplemental_file.pdf]

Supporting Information

Engineering surface framework TiO_6 single sites for unprecedented deep oxidative desulfurization

Shen Yu[1], Zhan Liu[1,2], Jia-Min Lyu[1], Chun-Mu Guo[1], Xiao-Yu Yang[1], Peng Jiang[1], Yi-Long Wang[3], Zhi-Yi Hu[1,2], Ming-Hui Sun[1], Yu Li[1], Li-Hua Chen[1,*] and Bao-Lian Su[1,4,*]

[1]Laboratory of Living Materials at the State Key Laboratory of Advanced Technology for Materials Synthesis and Processing, Wuhan University of Technology, Wuhan 430070, China;

[2]Nanostructure Research Center, Wuhan University of Technology, Wuhan 430070, China;

[3]School of Chemistry, Chemical Engineering and Life Science, Wuhan University of Technology, Wuhan 430070, China;

[4]Laboratory of Inorganic Materials Chemistry, University of Namur, Namur B-5000, Belgium

*__Corresponding authors.__ E-mails: chenlihua@whut.edu.cn; bao-lian.su@unamur.be

Experimental and Characterization Details

Preparation of Meso-TS-C

The mesoporous titanium silicate (Meso-TS-C, C represents cetyltrimethylammonium bromide) with a feeding Si/Ti ratio of 25 was prepared according to a modified procedure reported by a previous literature.[1] Typically, 5.49 g of diethylamine (DEA, Aladdin, AR) was dissolved into 108 g of diluted water, followed by adding 2.73 g of cetyltrimethylammonium bromide (CTAB, Aladdin, AR). The solution was vigorously stirred for 30 min. Hereafter, 0.68 g of tetrabutyl titanate (TBT, Aladdin, AR) was dissolved into 10.42 g of tetraethyl orthosilicate (TEOS, Aladdin, AR) to form a homogeneous mixture. The mixture was dropwise added into the above solution. After a vigorous stirring for 4 h, hydrothermal process was conducted in a Teflon-lining sealed by a stainless-steel autoclave at 100 ℃ for 2 days. Finally, the obtained powder was collected by filtering, washing by deionized water as well as alcohol, and drying at 120 ℃ for overnight. The Meso-TS-C was obtained after being calcined at 550 ℃ (ramp rate = 5 ℃/min) for 6 h in a muffle furnace.

Preparation of Meso-TS-3H and 6H

The mesoporous titanium silicates (Meso-TS-3H and 6H) were synthesized by a procedure similar to that of the Meso-TS-C, except that H_2O_2 was introduced into precursors. Typically, 5.49 g of DEA was dissolved into 108 g of diluted water, followed by adding 2.73 g of CTAB. The solution was vigorously stirred for 30 min. Hereafter, 0.68 g of TBT was dissolved into 10.42 g of TEOS to form a homogeneous mixture. The mixture was dropwise added into the above solution. After a vigorous stirring for 4 h, a H_2O_2 solution with a mass of 3 or 6 times as much as TBT added was introduced and the stirring was kept for another 30 min. The hydrothermal process was also conducted in a Teflon-lining sealed by a stainless-steel autoclave at 100 ℃ for 2 days. Finally, the obtained powder was collected by filtering, washing by deionized water as well as alcohol, and drying at 120 ℃ for overnight. The product was obtained after calcination at 550 ℃ (ramp rate = 5 ℃/min) for 6 h in a muffle furnace. According to the mass ratio of H_2O_2 solution to TBT, the obtained mesoporous titanium silicates were denoted as Meso-TS-3H or Meso-TS-6H.

Preparation of Nano-TS-1 with framework TiO_4 on the surface

The nanosized Titanium Silicalite-1 (TS-1) zeolite (Nano-TS-1) with a feeding Si/Ti ratio of 25 was synthesized. Typically, 0.68 g of TBT and 10.42 g of TEOS was mixed homogeneously. The mixture was added dropwise into 25.42 g of tetrapropylammonium hydroxide solution (TPAOH, 1 M in water, Aladdin) under stirring for 5 min. After vigorous stirring for 30 min, 10.45 g of deionized water were added in to form a clear solution with a molar composition of SiO_2: TiO_2: TPAOH: H_2O = 1: 0.04: 0.5: 35. Crystallization process was conducted in a Teflon-lining sealed by a stainless-steel autoclave at 180 ℃ for 3 days. Finally, the obtained powder was collected by filtering, washing by deionized water as well as alcohol, and drying at 120 ℃ for overnight. Nano-TS-1 zeolite was obtained after being calcined at 550 ℃ (ramp rate = 5 ℃/min) for 6 h in a muffle furnace.

Preparation of TS-TiO_4 containing only framework TiO_4

The TS-1 zeolite contains only framework TiO_4 (TS-TiO_4) was prepared according to a similar way as the Nano-TS-1 zeolite mentioned above. Herein, the feeding Si/Ti ratio was set to be 50 and the obtained powder was post-treated by washing with acid (1 M of HCl, solid/liquid ratio = 1 g/50 mL, stirring for 24 h at room temperature), filtering, washing by deionized water as well as alcohol, drying at 120 ℃ for overnight, and calcining at 550 ℃ (ramp rate = 5 ℃/min) for 6 h in a muffle furnace.

Preparation of Meso-TS-N with sole anatase on the pore surface

The Meso-TS-N (N represents nonionic surfactants) with a feeding Si/Ti ratio of 25 was synthesized according to a modified method reported by Ryoo’s group.[2] PEO-PPO-PEO (Pluronic P123, polyethylene oxide–polypropylene oxide–polyethylene oxide, average molecular mass of ~5800) was used as structure directing agent while TEOS was used as silicon source. In a typical synthesis, 4.5 g of concentrated HCl (Sinopharm Chemical Reagent Co., Ltd, AR, 36.0~38.0%) was added into 21.6 g of deionized water to form a homogeneous solution. 3.46 g of P123 was introduced into above solution with vigorous stirring under 45 ℃ for 6 h. Hereafter, a

homogeneous mixture of 0.5 g of TBT and 7.5 g of TEOS was added into this solution and the whole mixture was kept stirring at 45 ℃ for 24 h. A H_2O_2 solution with a mass of 6 times as TBT was introduced and the stirring was kept for another 30 min. Subsequently, the hydrothermal process was conducted under 100 ℃ for 24 h in a lining sealed by a stainless autoclave. Finally, the obtained powder was collected by filtering, washing by deionized water as well as alcohol, and drying at 120 ℃ for overnight. Meso-TS-N was obtained after calcination at 550 ℃ (ramp rate = 5 ℃/min) for 6 h in a muffle furnace.

Silylation post treatment of titanium silicate materials with hexadecyltrimethoxysilane ($(CH_3O)_3$-Si-C_{16})

The silylated titanium silicate materials (denoted as HD-Meso-TS-C and HD-Meso-TS-6H, HD represents hexadecyl groups) were synthesized by a post-silylation method reported by Xiao's group.[3] As a typical run for the synthesis of HD-Meso-TS-C, 0.5 g of the Meso-TS-C was dried at 160 ℃ under vacuum and then dispersed in 10 mL of anhydrous toluene by sonication at room temperature for 5 min. Then, 130 μL (C/Si = 0.04) of hexadecyltrimethoxysilane (Aladdin, AR) was dissolved within 20 mL of anhydrous toluene (Sinopharm Chemical Reagent Co., Ltd) and the titanium silicate material suspension was added to the solution under stirring. The mixture was stirred for 24 h at 500 rpm at room temperature. After filtering, washing with ethanol, and drying at 100 ℃ for overnight, the HD-Meso-TS-C was obtained. The HD-Meso-TS-6H was obtained according to a same procedure.

Catalyst characterizations

Power X-ray diffraction (XRD) patterns were recorded on a D8 ADVANCE with Cu Kα radiation (λ = 1.5413 Å) at a tube voltage of 40 kV and a tube current of 40 mA under ambient conditions. Data was collected at 2θ ranging from 5 ° to 65 ° with a step of 0.05 °/min.

Small-angle X-radiation diffraction (SAXD) were recorded on a Rigaku Ultimate IV with Cu Kα radiation (λ = 1.5413 Å) at a tube voltage of 40 kV and a tube current of 40 mA under ambient conditions. Data was collected at 2θ ranging from 0.5 ° to 10 ° with a step of 1 °/min.

The scanning electron microscopy (SEM) was collected on a Hitachi S-4800 scanning electron microscopy with extraction voltage of 5.0 kV and acceleration current of 10.0 μA.

The transmission electron microscope (TEM) and high-resolution TEM (HR-TEM) images were investigated by a JEOL JEM-2100F HRTEM, and the accelerating voltage was 200 kV. Scanning TEM (STEM)-energy-dispersive X-ray spectroscopy (EDX) spectrum was recorded with an EDX attached to HR-TEM. Elemental mapping results were acquired by the high-angle annular dark-field scanning transmission electron microscopy (HAADF-STEM) mode in combination with energy-dispersive X-ray spectroscopy (EDX) analysis.

N_2 adsorption–desorption isotherms were recorded using a Micromeritics ASAP 3020 gas sorptiometer after the samples were degassed at 300℃ under vacuum for 8 h. The mesopore surface area was determined from N_2 adsorption using the Brunauer-Emmett-Teller method. Using the Barret-Joyner-Halenda model, mesopore volume and mesopore size were determined by the desorption branches of N_2 isotherms. Total pore volumes were estimated from the adsorbed amount at a relative pressure P/P_0 of 0.99.

A Lambda 750 S equipment was applied to obtain Ultraviolet-visible (UV-vis) diffuse reflectance spectrum in the wavelength ranging from 800 nm to 200 nm with $BaSO_4$ as the reference sample.

Raman Ultraviolet resonance Raman spectroscopy (UV-Raman) spectrum was recorded on a Horiba Evolution spectrometer using the 325 nm line of a He-Cd laser as the excitation source.

X-ray photoelectron spectroscopy (XPS) was performed on Thermo Scientific K-Alpha+ (Thermo Fisher) spectrometer equipped with a mono Al-Kα X-ray source (excitation energy = 1486.6 eV). Spectrum curve fitting was carried out by using the XPSPEAK41 software.

Inductively coupled plasma optical emission spectrometer (ICP-OES, Agilent 730) was used to determine the element fraction of samples.

The nuclear magnetic resonance (NMR) spectra were recorded at room temperature using a JEOL ECZ-R spectrometer operating at 14.1 T (^{29}Si frequency = 119.2 MHz) equipped with a 3.2 mm AUTOMAS probe. The samples were packed in 3.2 mm zirconia rotors and spun at 10 kHz. The number of transients was 12000 for the ^{29}Si cross polarization (CP)-MAS spectra. The

relaxation delay was set to 300 s in ^{29}Si MAS spectra. The contact time was 5 ms ^{29}Si CP-MAS spectra. SPINAL-64 ^{1}H decoupling with an rf-field of 88 kHz was applied during the FID.

Ti K-edge analysis was conducted on the BL11B beamlines at the Shanghai Synchrotron Radiation Facility (SSRF). Before testing, samples were pressed into thin sheets with a diameter of 1 cm and sealed with Kapton film. The X-ray Absorption Fine Structure (XAFS) spectra were recorded using a 4-channel Silicon Drift Detector (SDD, Bruker 5040). Ti K-edge extended X-ray absorption fine structure (EXAFS) spectra were recorded using transmission mode. The XAFS spectra of these reference samples (Ti foil and Anatase) were obtained from a standard database recorded in transmission mode. The spectra were processed and analyzed by the software of Athena and Artemis.[4]

Computational details

Calculations were carried out using the Gaussian 09 program suite[5] based on density functional theory methods. B3LYP/6-31G(d, p) calculations[6,7] are performed for geometry optimization and frequency calculation. The empirical dispersion correction of Grimme for DFT-D calculations has been used. All minima were characterized by the lack of imaginary frequencies whereas only one imaginary frequency was identified for transition-state structures, which was associated to the normal mode of vibration connecting reactants and products. The energies of each steps were calculated as **initial Ti site:** G(R-Ti-OH); **Step 1: R-Ti-OH + Th -> R-Ti-OH-Th**, ΔG1 = G(R-Ti-OH-Th) – G(R-Ti-OH)-G(Th); **Step 2: R-Ti-OH-Th -> R-Ti-Th-H_2O**, ΔG2 = G(R-Ti-Th-H_2O) – G(R-Ti-OH-Th); **Transition state 1 (TS1): Step 1 -> Step 2 Dehydration energy barrier**, ΔG12 = G(TS1) – G(R-Ti-OH-Th); **Step 3: R-Ti-Th-H_2O +Dt -> R-Ti-Th-H_2O-Dt**, ΔG3 = G(R-Ti-Th-H_2O-Dt) – G(R-Ti-Th-H_2O) – G(Dt); **Step 4: R-Ti-Th-H_2O-Dt -> R-Ti-Th-H_2O-O-Dt**, ΔG4 = G(R-Ti-Th-H_2O-O-Dt) – G(R-Ti-Th-H_2O-Dt); **Transition state 2 (TS2): Step 3 -> Step 4 Oxidative desulfurization energy barrier**, ΔG34 = G(TS2) – G(R-Ti-Th-H_2O-Dt); **Step 5: R-Ti-Th-H_2O-O-Dt -> R-Ti-ThOv-H_2O + O-Dt**, ΔG5 = G(R-Ti-ThOv-H_2O +O-Dt) – G(R-Ti-Th-H_2O-O-Dt); **Step 6: R-Ti-ThOv-H_2O -> R-Ti-OH+Tb**, ΔG6 = G(R-Ti-OH+Tb) – G(R-Ti-ThOv-H2O); **Transition state 3 (TS3): Step 5 -> Step 6 Water splitting energy barrier**, ΔG56 = G(TS3) – G(R-Ti-ThOv-H_2O). Th, Dt, and Tb represent

tert-butyl hydroperoxide, 4,6-dimethyldibenzothiophene, and tert-butanol, respectively. The specific energy values are summarized and shown in **Table S1**.

Catalytic tests

Oxidative desulfurization (ODS): A certain amount of benzothiophene (BT), dibenzothiophene (DBT), and 4,6-dimethyldibenzothiophene (DMDBT) was dissolved in n-octane to act as a model fuel (decane or dodecane were used as internal standards). The concentration of sulfur in each model fuel was 1000, 1000, and 500 ppm for BT, DBT, and DMDBT, respectively. The reaction was performed in a 25 mL two-neck glass flask in an oil bath under vigorous stirring (600 rpm). In a typical run, 10 mL of model fuel and 20 mg of catalyst were added to the flask. Then, the temperature of the mixture was raised to a certain value and kept to be stable. Tert-butylhydroperoxide (TBHP, Sinopharm Chemical Reagent Co., Ltd, AR, 65 wt%) was used as an oxidant and the molar ratio of TBHP to sulfur in model fuel was fixed as 2. That is 31, 23, and 10 μL for the ODS of BT, DBT, and DMDBT, respectively. After adding TBHP into the mixture, the reaction was triggered and kept for a certain time. A small aliquots (~200 μL) of the reaction solution were extracted as a function of time through a 0.22 μm syringe filter (to remove suspended catalyst and stop the reactions from proceeding). All the organic components were determined quantitively by a gas chromatography (GC 7890B) equipped with a flame-ionization detector. The reactant conversion (X) is calculated by **Equation (1)** as following:

$$X = \left(1 - \frac{\frac{A_R}{A_I}}{\frac{A_{R0}}{A_{I0}}}\right) \times 100\% \qquad \textbf{Equation (1)}$$

A_R, A_I, A_{R0}, and A_{I0} represents the peak area of reactant, internal standard, initial reactant, initial internal standard determined by GC, respectively.

Recyclability: Catalysts were collected after every reaction cycle from reaction solutions, washed, and dried at 120℃ for overnight. And then, the dried catalysts were calcined at 550℃ for 6 h in air. The refreshed catalysts (recovery ratio > 95 wt% for every time) were reused in the next reaction cycle with the same ratio of reactant mixture: TBHP: catalyst: model fuel as the 1st reaction cycle.

Supporting Figures and Tables

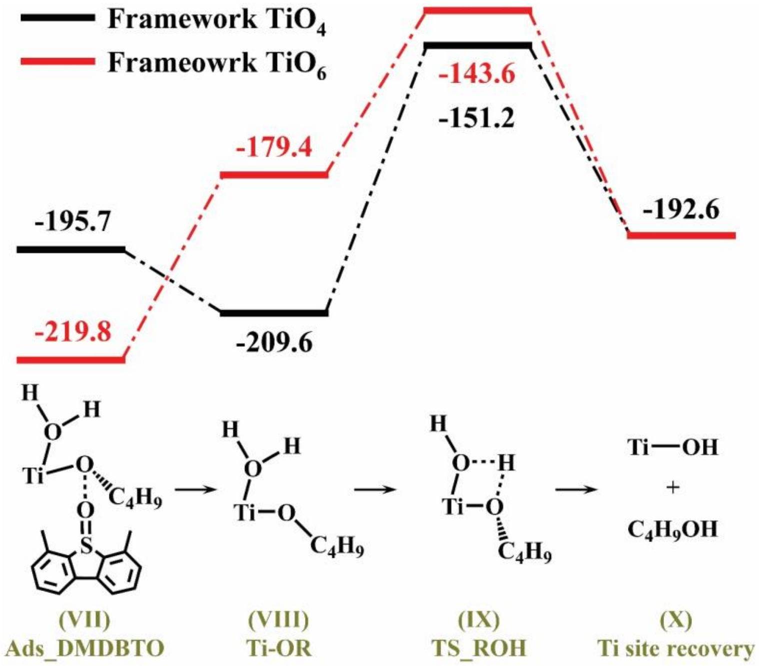

Figure S1. Reaction pathway and corresponding calculated energies for the ODS reaction over a framework TiO_4 site and a framework TiO_6 site, including ten steps, Steps VII–X of which is shown here. (Step VII) Ads_DMDBTO: the formation of oxide product DMDBTO; (Step VIII) Ti-OR: DMDBTO desorbs to form Ti-OR; (Step IX) TS_ROH: the transition state of the formation of ROH; (Step X) Ti site recovery: ROH desorbs to recover Ti site. R represents $C(CH_3)_3$ and all energies are given in kJ/mol. Steps I–VII are displayed in **Figure 1**.

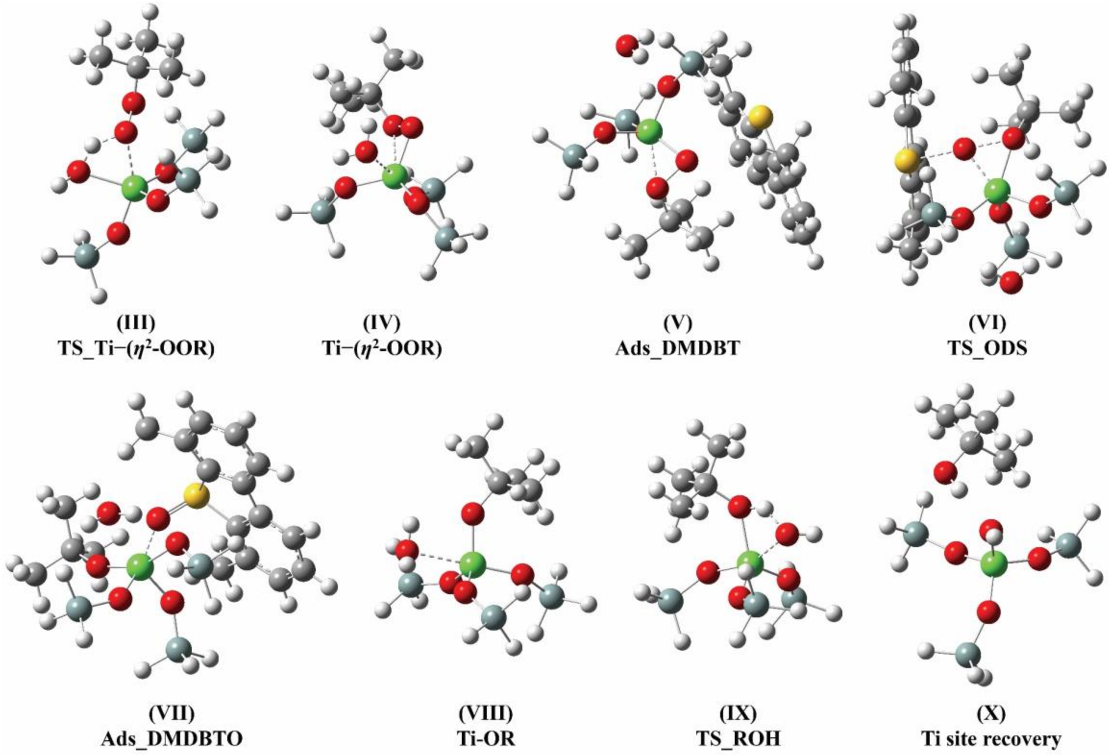

Figure S2. Calculated structures of the state of each step in the reaction pathway, including ten steps, Steps III–X over a framework TiO_4 site of which are shown here. (Step III) TS_Ti-(η^2-OOR): the transition state of an active intermediate; (Step IV) Ti-(η^2-OOR): the active intermediate; (Step V) Ads_DMDBT: the adsorption of a DMDBT molecule; (Step VI) TS_ODS: the transition state of the ODS reaction; (Step VII) Ads_DMDBTO: the formation of oxide product DMDBTO; (Step VIII) Ti-OR: DMDBTO desorbs to form Ti-OR; (Step IX) TS_ROH: the transition state of the formation of ROH; (Step X) Ti site recovery: Ti site is recovered by the desorption of ROH. R represents $C(CH_3)_3$ and calculated structures of the state of Steps I–II are shown in **Figure 1**.

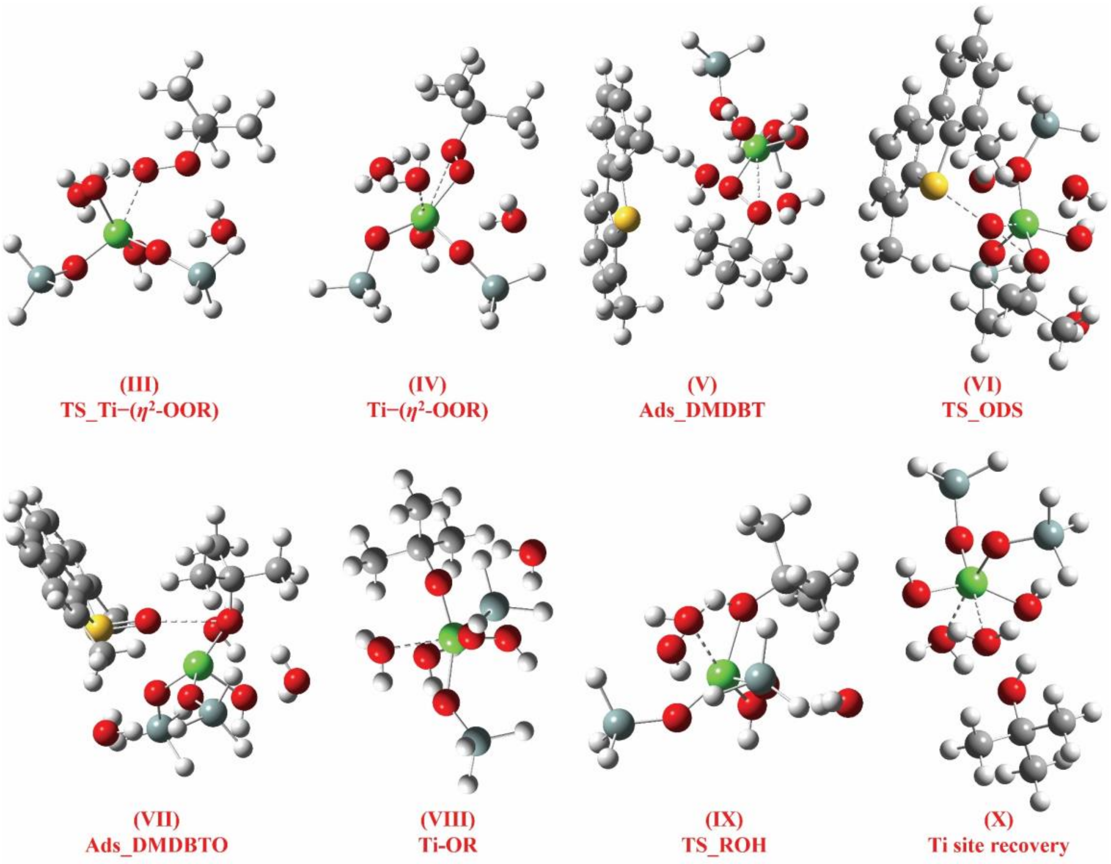

Figure S3. Calculated structures of the state of each step in the reaction pathway, including ten steps, Steps III–X over a framework TiO_6 site of which are shown here. (Step III) TS_Ti-(η^2-OOR): the transition state of an active intermediate; (Step IV) Ti-(η^2-OOR): the active intermediate; (Step V) Ads_DMDBT: the adsorption of a DMDBT molecule; (Step VI) TS_ODS: the transition state of the ODS reaction; (Step VII) Ads_DMDBTO: the formation of oxide product DMDBTO; (Step VIII) Ti-OR: DMDBTO desorbs to form Ti-OR; (Step IX) TS_ROH: the transition state of the formation of ROH; (Step X) Ti site recovery: Ti site is recovered by the desorption of ROH. R represents -$C(CH_3)_3$ and calculated structures of the state of Steps I–II are shown in **Figure 1**.

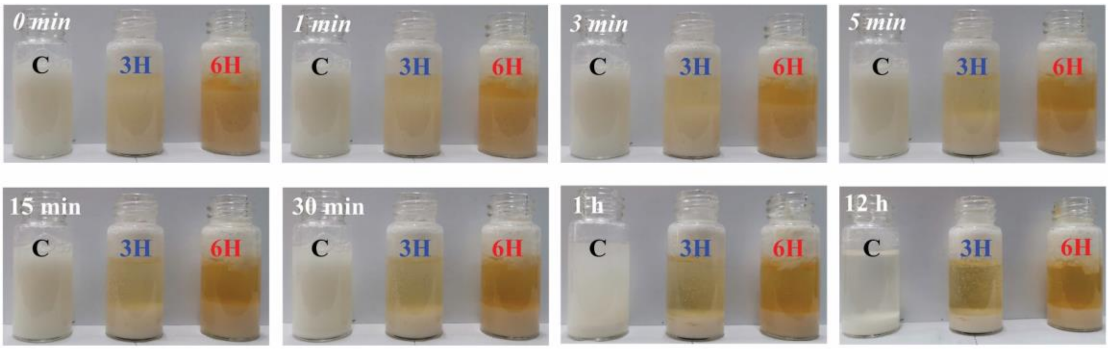

Figure S4. Optical photographs showing the precipitation process of the mother liquid of the Meso-TS-C, Meso-TS-3H, and Meso-TS-6H.

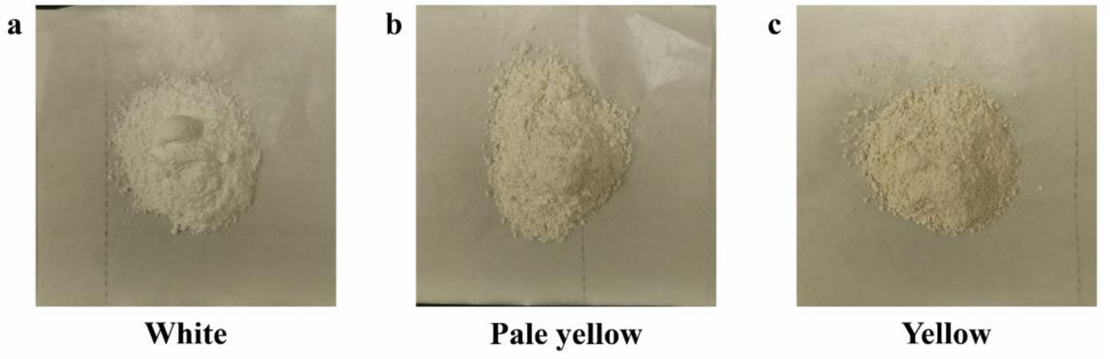

Figure S5. Optical photographs showing the color of intermediate product powders of the (a) Precursor-C, (b) Precursor-3H, and (c) Precursor-6H before the final calcination.

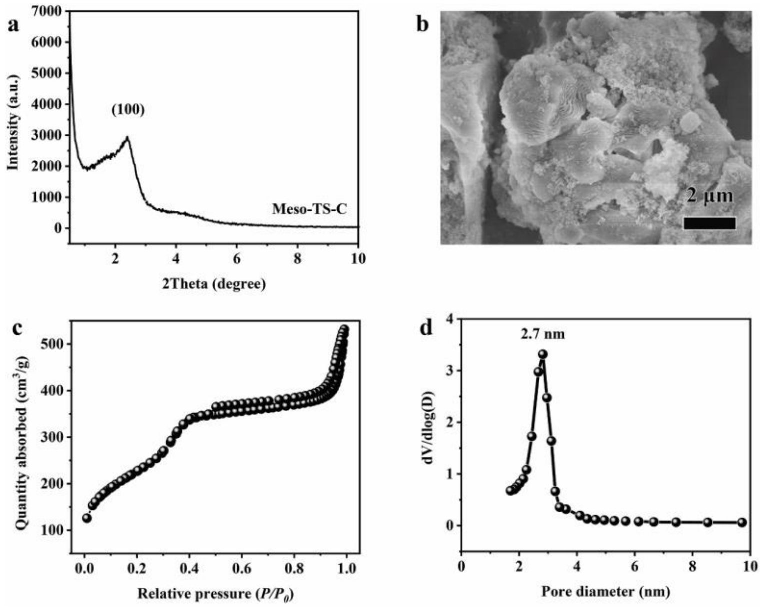

Figure S6. Characterizations of the conventional Meso-TS-C: (a) SAXD pattern, (b) SEM image, (c) N_2 adsorption–desorption isotherm and (d) pore size distribution.

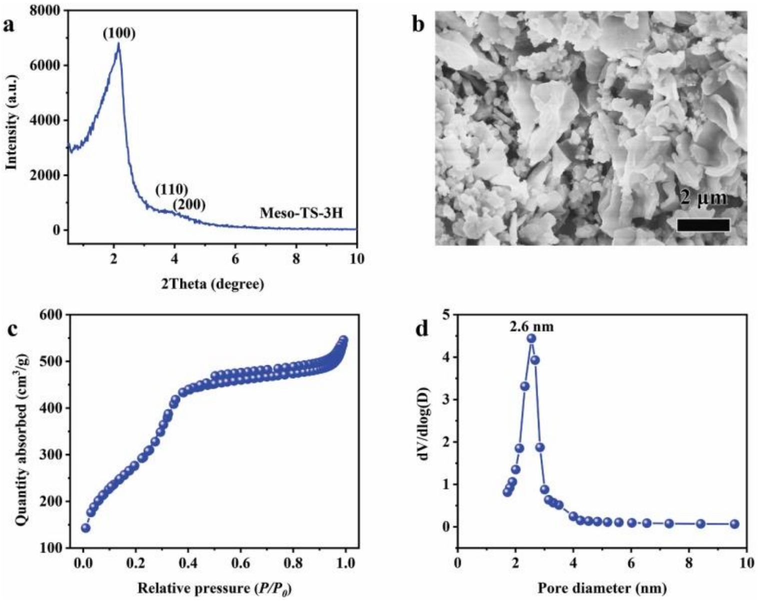

Figure S7. Characterizations of the Meso-TS-3H synthesized by engineering the electrostatic interface: (a) SAXD pattern, (b) SEM image, (c) N_2 adsorption–desorption isotherm and (d) pore size distribution.

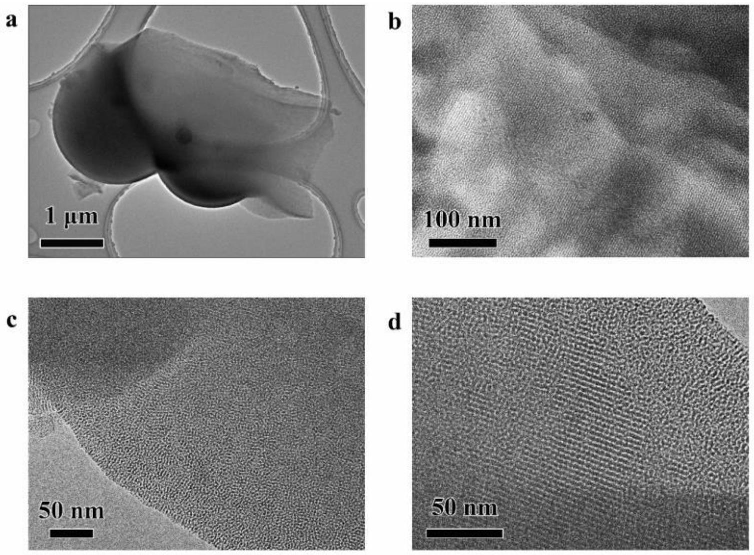

Figure S8. (a) TEM image and (b–d) high-resolution TEM images of the Meso-TS-6H.

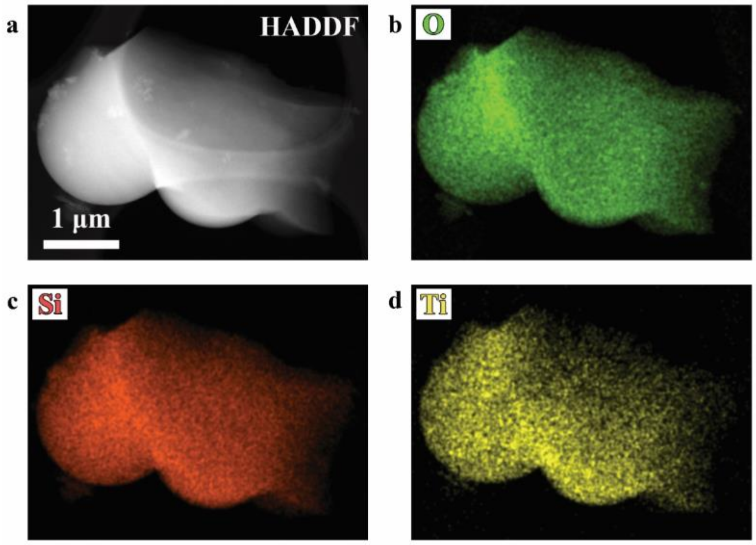

Figure S9. HADDF-STEM-EDX mappings of the Meso-TS-6H: (a) HADDF image, (b–d) element distribution of O, Si, and Ti, respectively.

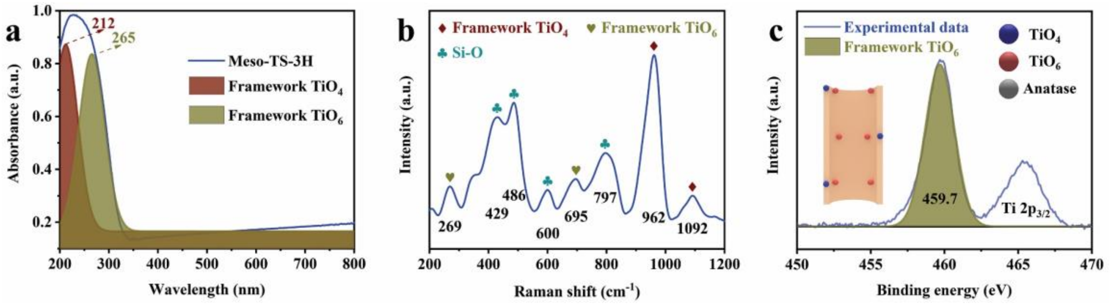

Figure S10. Characterizations of Ti state in the Meso-TS-3H: (a) UV-vis spectra, (b) Raman spectra, and (c) XPS spectra of Ti $2p_{3/2}$. The signal at ~466 eV in the XPS spectra of (c) is assigned to Ti $2p_{1/2}$.

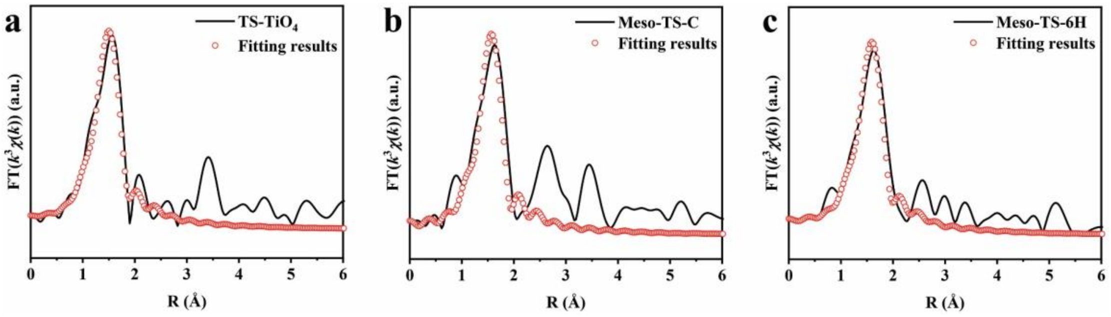

Figure S11. Fitting results of the FT *R*-space Ti K-edge EXAFS of (a) TS-TiO_4, (c) Meso-TS-C, and (c) Meso-TS-6H. The data are K^3-weighted and not phase corrected.

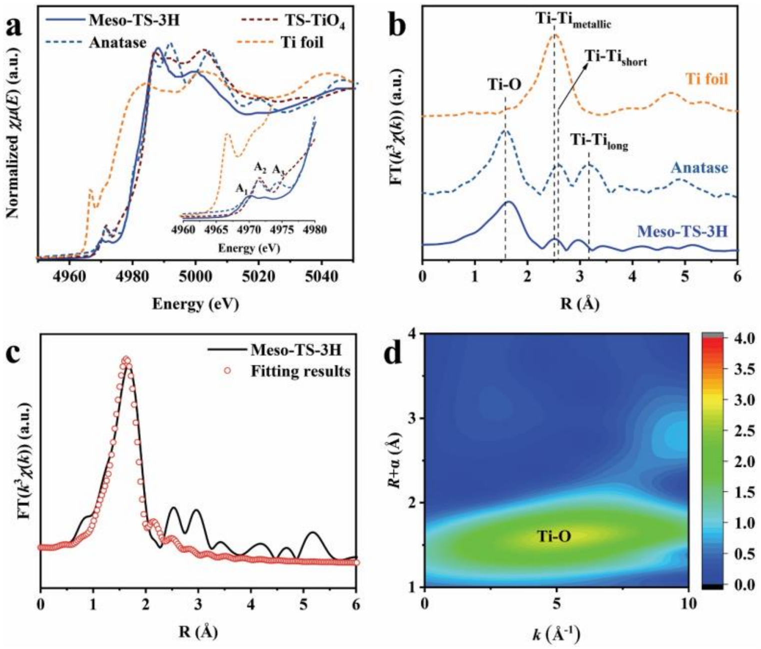

Figure S12. Characterizations of the state of Ti species in the Meso-TS-3H**.** (a) Ti K-edge XANES spectra, (b) Fourier-transform (FT) EXAFS spectra, (c) fitting results of the FT *R*-space Ti K-edge EXAFS (the data are K^3-weighted and not phase corrected), and (d) Wavelet transform for the k^3-weighted EXAFS of the Meso-TS-C.

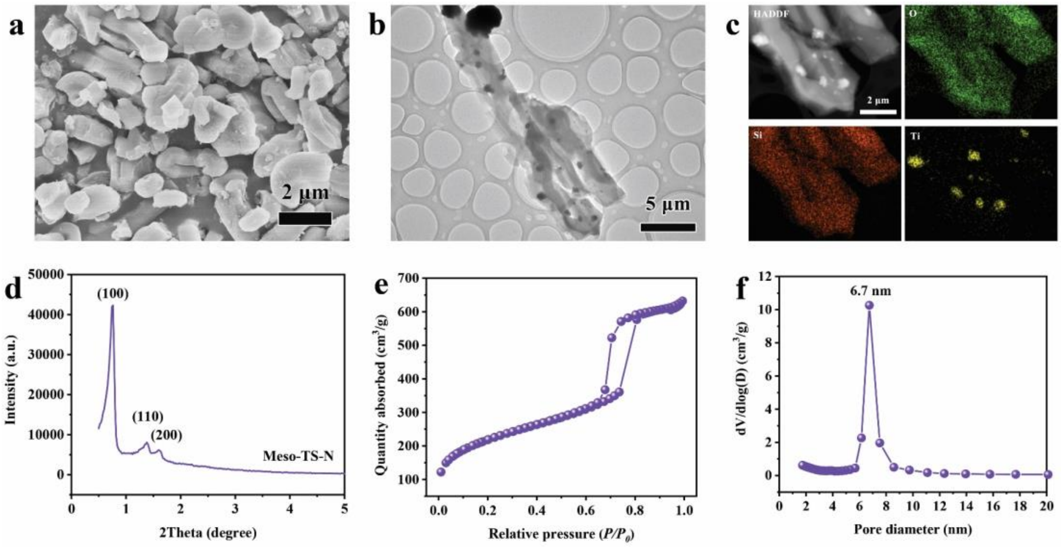

Figure S13. Characterizations of Meso-TS-N: (a) SEM image, (b) TEM images, (c) HADDF and mapping images, (d) SAXD pattern, (e) N_2 adsorption–desorption isotherm, and (f) pore size distribution.

SEM images (**Figure S13a**) shows that the Meso-TS-N possesses a worm-like morphology. TEM images and mapping results shows the existence of anatase particles as aggregations of Ti elements in Meso-TS-N (**Figure S13b and c**). The SAXD pattern (**Figure S13d**) of the Meso-TS-N shows typical diffraction peaks belong to the (100), (110), and (200) faces of SBA-15 structure, indicating the existence of highly ordered mesopores. The N_2 adsorption–desorption istherm (**Figure S13e**) also evidences the existence of abundant mesopores by showing a typical type IV isotherm with a obvious hysteresis loop. The mesopore diameter is determined to be 6.7 nm for the Meso-TS-N (**Figure S13f**).

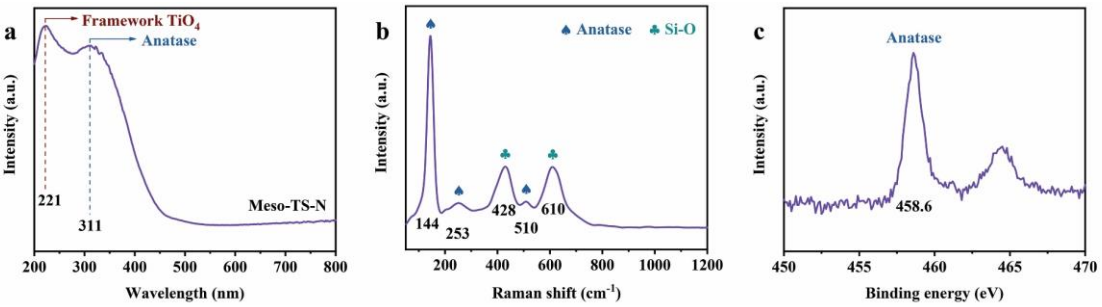

Figure S14. Characterizations of Ti state in the Meso-TS-N: (a) UV-vis spectra, (b) Raman spectra, and (c) XPS spectra of Ti $2p_{3/2}$. The signal at ~466 eV in the XPS spectra of (c) is assigned to Ti $2p_{1/2}$.

Adsorption peaks at 221 and 311 nm in UV-vis spectrums are attributed to framework tetrahedrally coordinated Ti (TiO_4) and anatase phase, respectively (**Figure S14a**).[8] Raman bands at 144 and 510 cm^{-1} are attributed to anatase phase while the rest of peaks are assigned to Si-O structure (**Figure S14b**).[9] For XPS results, peaks at 458.6 eV are attributed to anatase phase (**Figure S14c**).[10] Collectively, **Figure S14** shows that the Meso-TS-N possesses framework TiO_4 and anatase in the pore wall while sole anatase phase on the pore surface.

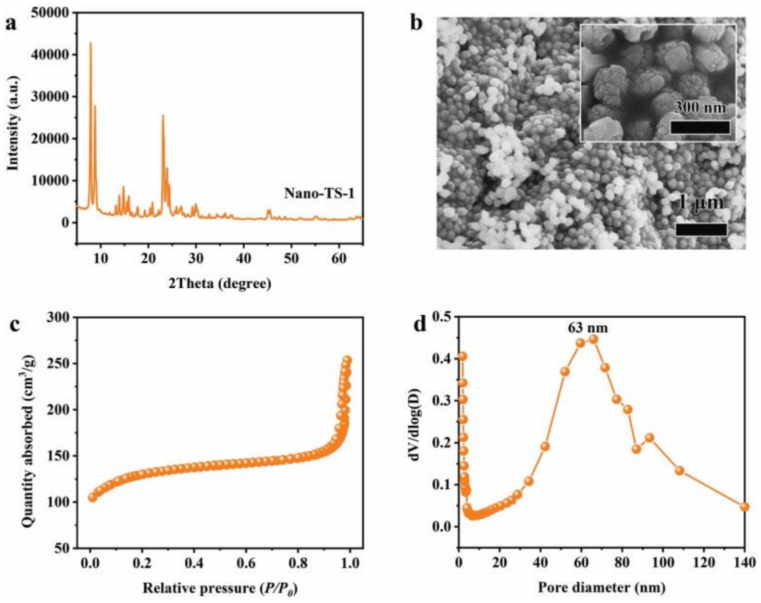

Figure S15. Characterizations of the Nano-TS-1: (a) XRD pattern, (b and the inset) SEM images, (c) N_2 adsorption–desorption isotherm, and (d) pore size distribution.

The Nano-TS-1 shows a typical MFI XRD pattern (**Figure S15a**) and berry-like crystal morphology (~200 nm) (**Figure 15b**). The N_2 adsorption–desorption isotherm (**Figure S15c**) of the Nano-TS-1 is a typical type IV isotherm without any hysteresis loop, indicating pure micropore structure. Some macropores centering at 63 nm are stacking voids of nanosized crystals (**Figure S15d**).

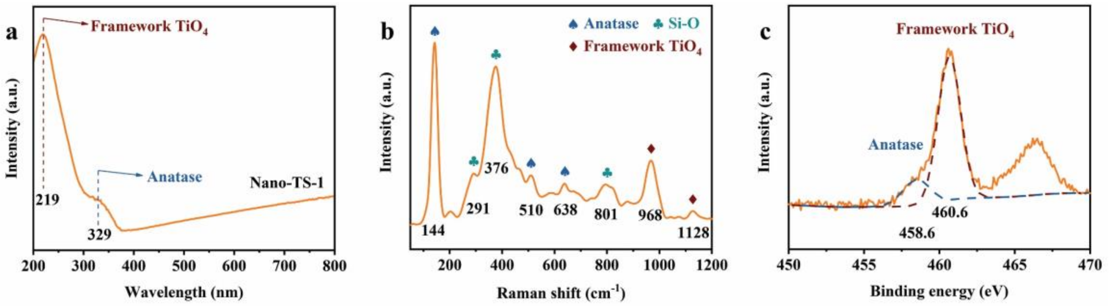

Figure S16. Characterizations of Ti state in the Nano-TS-1: (a) UV-vis spectra, (b) Raman spectra, and (c) XPS spectra of Ti $2p_{3/2}$. The signal at ~466 eV in the XPS spectra of (c) is assigned to Ti $2p_{1/2}$.

Adsorption peaks at 219 and 329 nm in UV-vis spectra are attributed to framework tetra-coordinated Ti (TiO_4) and anatase phase, respectively (**Figure S16a**).[8] Raman bands at 968 and 1128 cm^{-1} are attributed to framework TiO_4. Bands at 144 and 510 cm^{-1} are attributed to anatase phase. The rest of peaks locating at 291, 376, and 801 cm^{-1} are assigned to Si-O structure (**Figure S16b**).[9] For XPS results, peaks at 458.6 and 460.6 eV are attributed to anatase phase and Framework TiO_4, respectively (**Figure S16c**).[10] Collectively, **Figure S16** show that the Nano-TS-1 possesses framework TiO_4 and anatase in the crystal and on the surface.

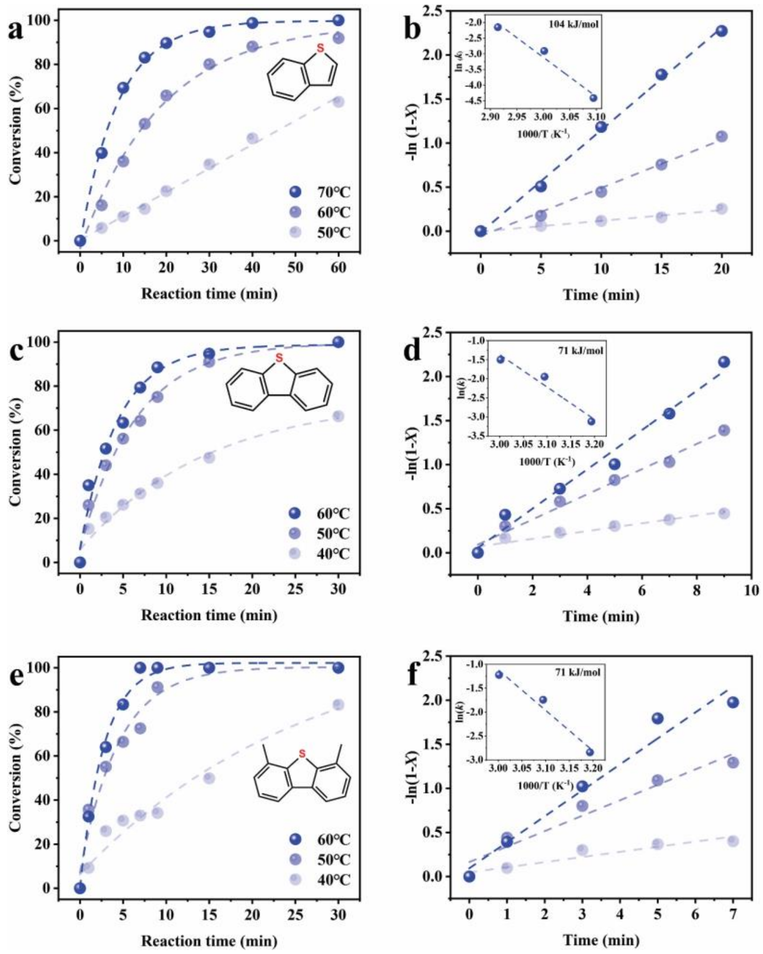

Figure S17. Oxidative desulfurization of (a, b) BT, (c, d) DBT, and (e, f) DMDBT over Meso-TS-3H: (a, c, e) reactant conversion as a function of reaction time at different reaction temperature and (b, d, f) corresponding Arrhenius plot and fitting results. Dash lines are fitting resullts.

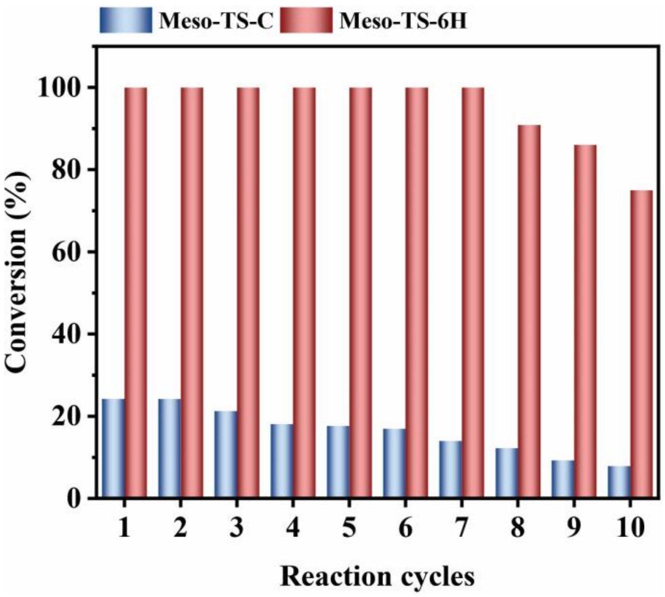

Figure S18. The reusability of Meso-TS-C and Meso-TS-6H during 10-times reaction cycles.

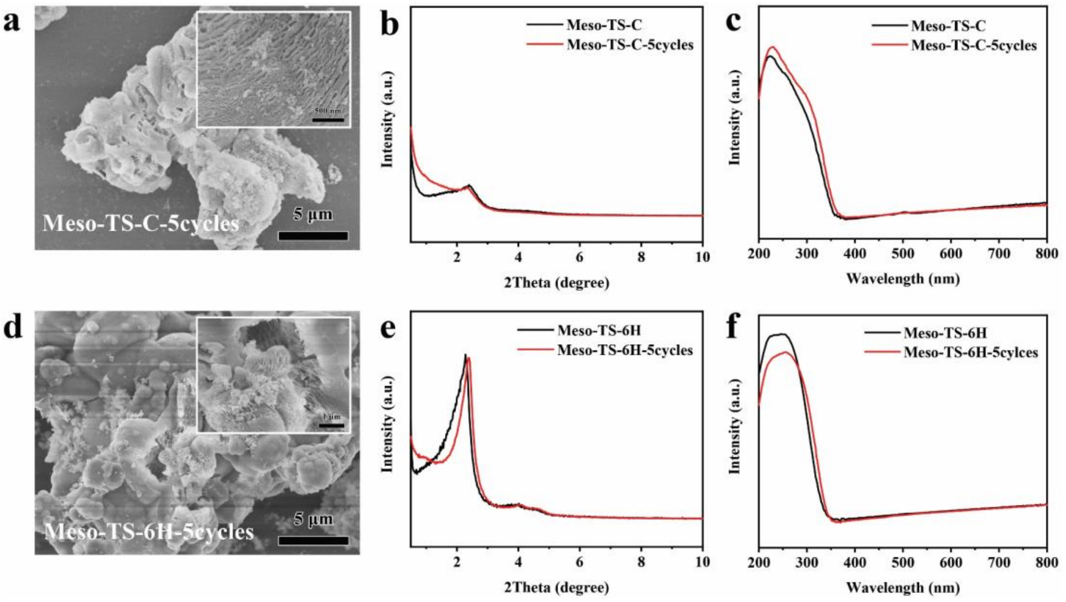

Figure S19. Characterization of (a–c) Meso-TS-C-5cycles and (d–f) Meso-TS-6H-5cycles after 5-times reaction cycles: (a, d) SEM images, (b, e) SAXD patterns, and (c, f) UV-vis spectra.

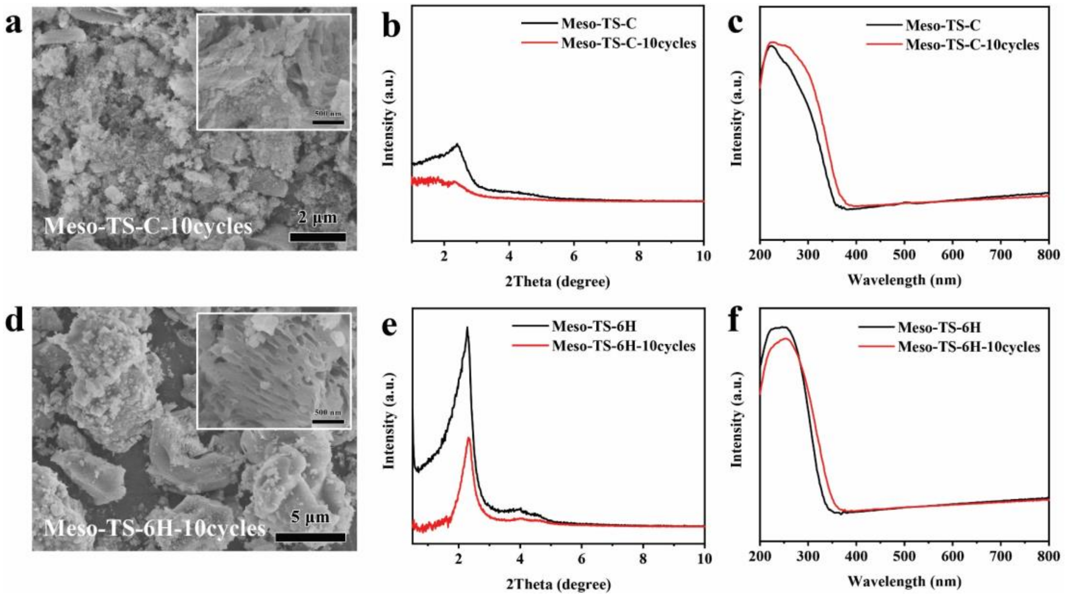

Figure S20. Characterization of (a–c) Meso-TS-C-10cycles and (d–f) Meso-TS-6H-10cycles after 10-times reaction cycles: (a, d) SEM images, (b, e) SAXD patterns, and (c, f) UV-vis spectra.

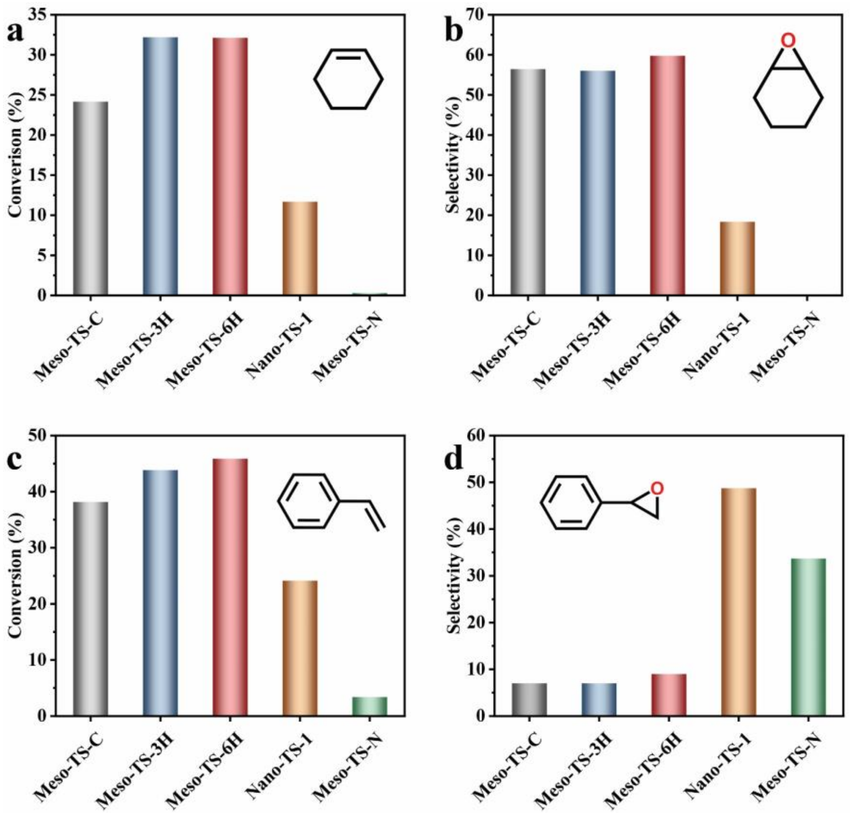

Figure S21. Catalytic performance over different catalysts: (a) cyclohexene conversion and (b) selectivity to epoxy cyclohexane, (c) styrene conversion and (d) selectivity to styrene oxide.

Table S1. The calculated energies of the states of each step in the ODS reaction pathway over a framework TiO_4 site and a framework TiO_6 site.

Value	Energy (Ha)	Energy (kJ/mol)	Plot Energy (Ha)	Plot Energy (kJ/mol)
**TiO_4**	0.000	0.00	0.0000	0.00
ΔG1	–0.0085	–22.24	–0.0085	–22.24
ΔG12	0.0163	42.69	0.0078	20.45
ΔG2	–0.0025	–6.49	–0.0109	–28.73
ΔG3	–0.0271	–71.10	–0.0380	–99.83
ΔG34	0.0203	53.50	–0.0176	–46.33
ΔG4	–0.0365	–95.88	–0.0745	–195.70
ΔG5	–0.0053	–13.87	–0.0798	–209.57
ΔG56	0.0222	58.36	–0.0576	–151.21
ΔG6	0.0065	16.94	–0.0734	–192.63
**TiO_6**	0.0000	0.00	0.0000	0.00
ΔG1	–0.0300	–78.68	–0.0300	–78.68
ΔG12	0.0209	54.76	–0.0091	–23.91
ΔG2	0.0079	20.72	–0.0221	–57.96
ΔG3	–0.0327	–85.94	–0.0548	–143.90
ΔG34	0.0292	76.61	–0.0256	–67.29
ΔG4	–0.0289	–75.86	–0.0837	–219.75
ΔG5	0.0154	40.31	–0.0683	–179.45
ΔG56	0.0136	35.82	–0.0547	–143.63
ΔG6	–0.0038	–10.05	–0.0722	–189.50

Table S2. Textural properties of different samples.

Sample	Si/Ti[a] (mol/mol)	Surface Ti content[b] (atomic %)	S_{BET}[c] (m^2g^{-1})	S_{micro} (m^2g^{-1})	V_{tol} (cm^3g^{-1})	V_{micro} (cm^3g^{-1})
**Meso-TS-C**	25	0.32	827	/	0.82	/
**Meso-TS-3H**	24	1.43	1269	/	0.79	/
**Meso-TS-6H**	24	1.47	1201	/	0.89	/
**Nano-TS-1**	42	0.44	393	228	0.39	0.13
**Meso-TS-N**	37	0.28	743	/	0.93	/

[a] Si/Ti ratio was determined by ICP-OES. [b] surface Ti content determined by XPS. [c] BET surface area and pore volume were obtained by analysis of N_2 adsorption–desorption data: BET surface area (**S_{BET}**), micropore BET surface area (**S_{micro}**), totol pore volume (**V_{tol}**), and micropore volume (**V_{micro}**).

Table S3. Curvefit parameters for Ti K-edge EXAFS for different samples.

Sample	Path	Coordination Number	R (Å)	σ^2 (Å^2)	ΔE_0 (eV)	R-factor
**TS-TiO$_4$**	Ti-O	3.1(0.5)	1.90(0.02)	0.008(0.002)	7(2)	0.016
**Meso-TS-C**	Ti-O	3.3(0.8)	1.95(0.02)	0.006(0.003)	10(2)	0.017
**Meso-TS-3H**	Ti-O	3.9(0.6)	2.01(0.02)	0.008(0.003)	10(2)	0.013
**Meso-TS-6H**	Ti-O	4.2(0.7)	1.99(0.02)	0.008(0.003)	11(2)	0.014

R is bond distance; σ^2 is Debye-Waller factor (a measure of thermal and static disorder in absorber-scatterer distances); ΔE_0 is edge energy shift (the difference between the zero-kinetic energy value of the sample and that of the theoretical model). R-factor is used to value the goodness of the fitting.

Table S4. ODS performance reported by previous literatures and this work.

ODS	Catalyst	Mass of catalyst (mg)	Removal content	Reaction time	reference
**BT**	**Meso-TS-3H**	**20**	**920 ppm**	**60 min**	**This work**
	**Meso-TS-6H**	**20**	**910 ppm**	**60 min**	**This work**
	Hierarchical TS-1	12.5	45 ppm	~60 min	Applied Catalysis B: Environmental, 2014, 146, 35–42[11]
	V/Beta	20	550 ppm	60 min	Applied Catalysis B: Environmental, 2022, 305, 121044[12]
**DBT**	**Meso-TS-3H**	**20**	**520 ppm**	**3 min**	**This work**
	**Meso-TS-6H**	**20**	**740 ppm**	**3 min**	**This work**
	Nanosized hierarchical TS-1	Si/Ti = 10	500 ppm	30 min	J. Mater. Chem. A, 2018, 6, 8757[13]
	V/Beta	20	~180 ppm	3 min	Applied Catalysis B: Environmental, 2022, 305, 121044[12]
	Hierarchical TS-1	12.5	~90 ppm	3 min	Applied Catalysis B: Environmental, 2014, 146, 35–42[11]
	Titanosilicate zeolite precursors (ATZ-TEA)	20	400 ppm	3 min	Chem. Sci., 2020, 11, 12341[14]
**4,6-DMDBT**	**Meso-TS-3H**	**20**	**500 ppm**	**7 min**	**This work**
	**Meso-TS-6H**	**20**	**500 ppm**	**3 min**	**This work**
	Nanosized hierarchical TS-1	Si/Ti = 10	500 ppm	30 min	J. Mater. Chem. A, 2018, 6, 8757[13]
	V/Beta	20	~500 ppm	~15 min	Applied Catalysis B: Environmental, 2022, 305, 121044[12]
	Hierarchical TS-1 (H-TS-1-50)	50	500 ppm	40 min	Journal of Colloid and Interface Science, 2022, 617, 32[15]

References

1. Lin W, Cai Q, Pang W *et al.* New mineralization agents for the synthesis of MCM-41. *Microporous Mesoporous Mater* 1999; **33**: 187–96.

2. Choi M, Heo W, Kleitz F *et al.* Facile synthesis of high quality mesoporous SBA-15 with enhanced control of the porous network connectivity and wall thickness. *Chem Commun* 2003: 1340–1.

3. Jin Z, Wang L, Zuidema E *et al.* Hydrophobic zeolite modification for in situ peroxide formation in methane oxidation to methanol. *Science* 2020; **367**: 193–7.

4. Ravel B, Newville M. ATHENA, ARTEMIS, HEPHAESTUS: data analysis for X-ray absorption spectroscopy using IFEFFIT. *J Synchrotron Radiat* 2005; **12**: 537–41.

5. Gaussian R, Trucks G, Schlegel H *et al.* Gaussian, Gaussian, Inc., Wallingford, CT. *Gaussian, Inc, Wallingford CT* 2004.

6. Stephens PJ, Devlin FJ, Chabalowski CF *et al.* Ab initio calculation of vibrational absorption and circular dichroism spectra using density functional force fields. *J Phys Chem* 1994; **98**: 11623–7.

7. Becke AD. Density‐functional thermochemistry. I. The effect of the exchange‐only gradient correction. *J Chem Phys* 1992; **96**: 2155–60.

8. Gordon CP, Engler H, Tragl AS *et al.* Efficient epoxidation over dinuclear sites in titanium silicalite-1. *Nature* 2020; **586**: 708–13.

9. Fan F, Feng Z, Li C. UV Raman spectroscopic studies on active sites and synthesis mechanisms of transition metal-containing microporous and mesoporous materials. *Acc Chem Res* 2010; **43**: 378–87.

10. Pang C, Xiong J, Li G *et al.* Direct ring C–H bond activation to produce cresols from toluene and hydrogen peroxide catalyzed by framework titanium in TS-1. *J Catal* 2018; **366**: 37–49.

11. Serrano DP, Sanz R, Pizarro P *et al.* Hierarchical TS-1 zeolite as an efficient catalyst for oxidative desulphurization of hydrocarbon fractions. *Appl Catal B Environ* 2014; **146**: 35–42.

12. Chen L, Ren J-T, Yuan Z-Y. Increasing the utilization of SiBeta support to anchor dual active sites of transition metal and heteropolyacids for efficient oxidative desulfurization of fuel. *Appl Catal B Environ* 2022; **305**: 121044.

13. Bai R, Sun Q, Song Y *et al.* Intermediate-crystallization promoted catalytic activity of titanosilicate zeolites. *J Mater Chem A* 2018; **6**: 8757–62.

14. Bai R, Navarro MT, Song Y *et al.* Titanosilicate zeolite precursors for highly efficient oxidation reactions. *Chem Sci* 2020; **11**: 12341–9.

15. Wang H, Du G, Chen S *et al.* Steam-assisted strategy to fabricate anatase-free hierarchical titanium silicalite-1 single-crystal for oxidative desulfurization. *J Colloid Interface Sci* 2022; **617**: 32–43.

Appendix: GC results

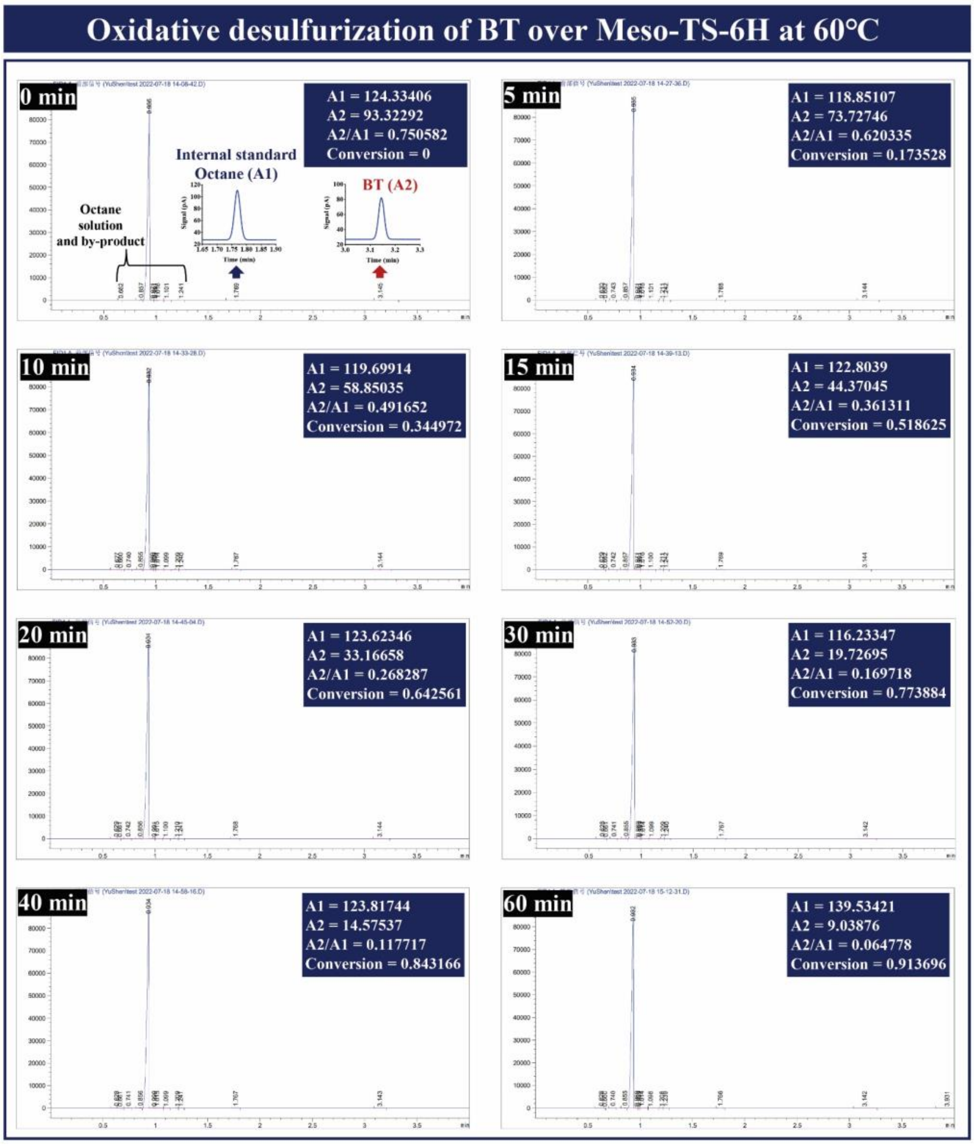

GC results of BT during ODS over Meso-TS-6H.

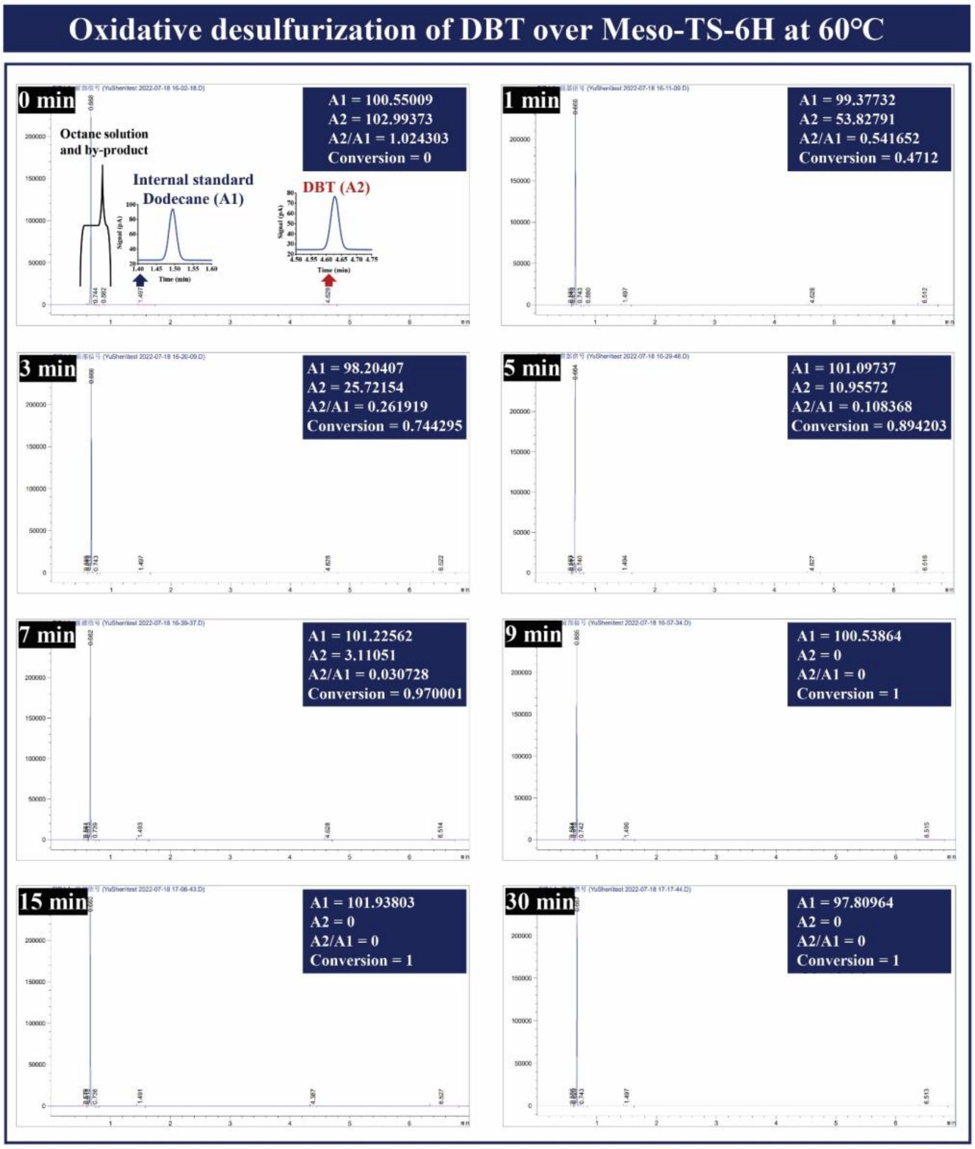

GC results of DBT during ODS over Meso-TS-6H.

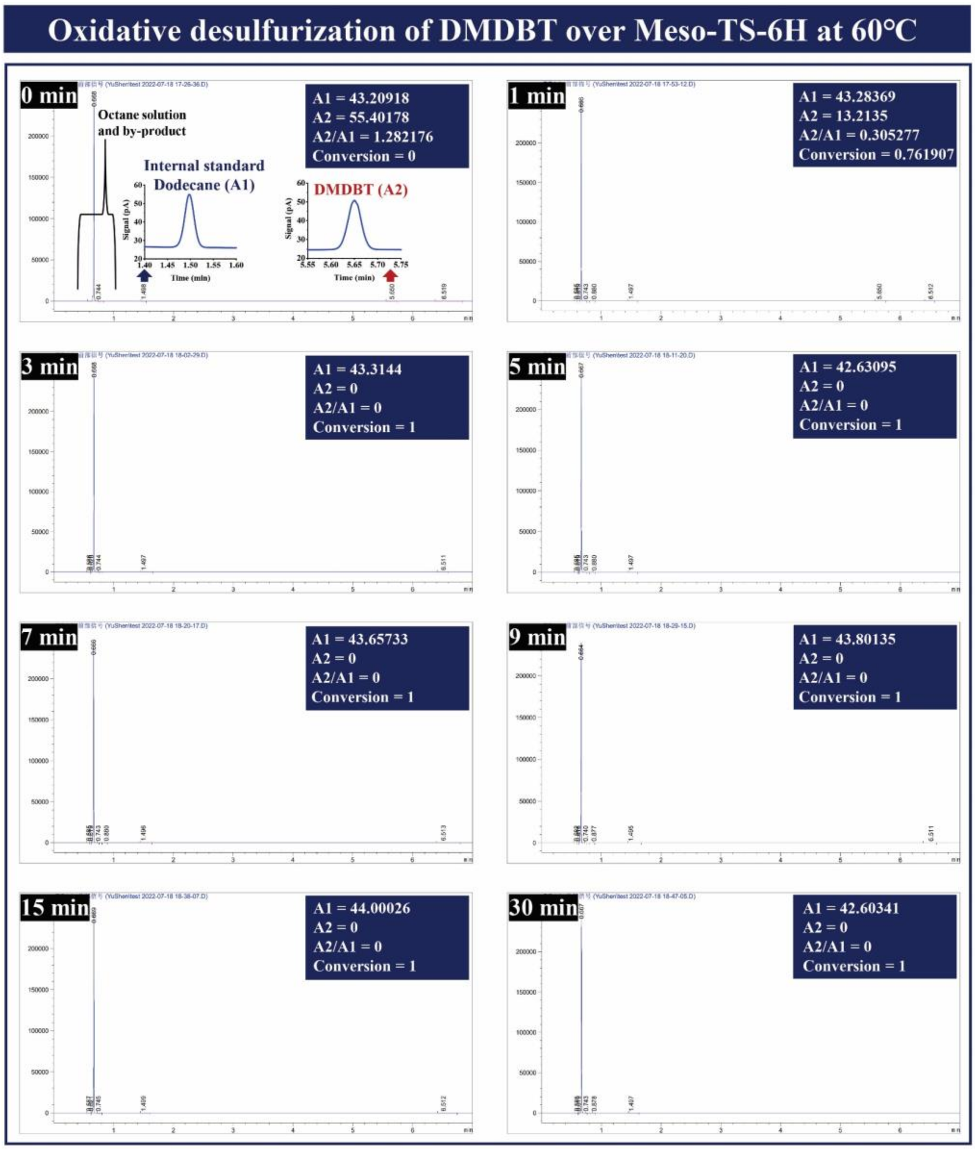

GC results of DMDBT during ODS over Meso-TS-6H.
